# Directionality in Aesthetic Judgments and Performance Evaluation: Sport Judges and Laypeople Compared

**DOI:** 10.3389/fpsyg.2017.02109

**Published:** 2017-12-05

**Authors:** Florian Loffing, Stefanie Nickel, Norbert Hagemann

**Affiliations:** ^1^Institute of Sport Science, University of Oldenburg, Oldenburg, Germany; ^2^Institute of Sports and Sports Sciences, University of Kassel, Kassel, Germany

**Keywords:** laterality, scanning habit, fluency, preference, bias, expertise, gymnastics

## Abstract

Left-to-right readers are assumed to demonstrate a left-to-right bias in aesthetic preferences and performance evaluation. Here we tested the hypothesis that such bias occurs in left-to-right reading laypeople and gymnastic judges (*n* = 48 each) when asked to select the more beautiful image from a picture pair showing gymnastic or non-gymnastic actions (Experiment 1) and to evaluate videos of gymnasts’ balance beam performances (Experiment 2). Overall, laypeople demonstrated a stronger left-to-right bias than judges. Unlike judges, laypeople rated images with left-to-right trajectory as more beautiful than content-wise identical images with right-to-left trajectory (Experiment 1). Also, laypeople tended to award slightly more points to videos showing left-to-right as opposed to right-to-left oriented actions (Experiment 2); however, in contrast to initial predictions the effect was weak and statistically unreliable. Collectively, judges, when considered as a group, seem less prone to directional bias than laypeople, thus tentatively suggesting that directionality may be an issue for *un*skilled but not for *skilled* judging. Possible mechanisms underlying the skill effect in Experiment 1 and the absence of clear bias in Experiment 2 are discussed alongside propositions for a broadening of perspectives in future research.

## 1. Introduction

Asymmetry in scanning habit established through reading and writing in one particular direction over years has been proposed as one mechanism eliciting directional bias in tasks (un)related to reading and writing (Kazandjian and Chokron, 2008; Vaid, 2011; Karim et al., 2016). For example, a bias in the direction of reading/writing habit has been found in the spatial representation of actions (Maass and Russo, 2003; Dobel et al., 2007), line bisection (Chokron and Imbert, 1993), aesthetic preferences (Nachson et al., 1999; Chokron and De Agostini, 2000; Ishii et al., 2011), performance evaluation (Maass et al., 2007) and the perception of motion (Morikawa and McBeath, 1992) or speed (Szego and Rutherford, 2008).

With regard to aesthetic preferences, left-to-right readers tend to perceive a stimulus (e.g., picture, painting, landscape photograph) with left-to-right orientation and/or the region of interest or weight on the right side as more beautiful than a horizontally mirrored version of the same stimulus (e.g., Christman and Pinger, 1997; Nachson et al., 1999; Chokron and De Agostini, 2000; Ishii et al., 2011; Treiman and Allaith, 2013; Friedrich et al., 2014). To test for directionality in aesthetic preferences, a common methodological approach is to present a vertically aligned pair of pictures that are identical in content but vary in horizontal orientation only (i.e., original and mirrored). Participants are then asked to select the picture from the pair that they think looks aesthetically more pleasant or beautiful. Using such methodology and counterbalancing the vertical position of pictures with left-to-right and right-to-left content within pairs, left-to-right reading individuals were shown to preferentially identify pictures with a left-to-right orientation as aesthetically more pleasant or beautiful. By contrast, research including right-to-left readers reported aesthetic preference in opposite direction (e.g., Nachson et al., 1999; Chokron and De Agostini, 2000; Ishii et al., 2011). Findings from this research highlight the important role of scanning habit and can be seen in line with the processing fluency theory of aesthetic pleasure, which basically states that “the more fluently the perceiver can process an object, the more positive is his or her aesthetic response” (Reber et al., 2004, p. 365).

In some studies, however, right-to-left readers demonstrated, on average, a left-to-right bias of smaller (Friedrich et al., 2014) or even similar (Treiman and Allaith, 2013) magnitude compared to left-to-right readers. These findings indicate that basic mechanisms related to brain lateralisation, and right hemisphere specialization for visuospatial processing in particular, might make individuals generally prone to a left-to-right directional bias (Levy, 1976; Beaumont, 1985). In this regard, reading or writing habit could then be assumed to either reinforce (in left-to-right readers), attenuate or reverse (in right-to-left readers) that bias (Chokron, 2002). Overall, the evidence available so far suggests that left-to-right readers should demonstrate the strongest and most reliable directional bias (i.e., toward left-to-right). In the two experiments reported here, we focussed on left-to-right readers. In Experiment 1 (see section 3 below), we hypothesized that participants would preferentially choose the picture with left-to-right oriented content when asked to select the more beautiful image from a picture pair.

Directional bias in aesthetic preference also occurs when watching videos (Maass et al., 2007; Friedrich et al., 2014). Even more, Friedrich et al. (2014) reported that left-to-right readers demonstrated stronger left-to-right bias in their aesthetic judgments when they rated videos as opposed to pictures that showed screenshots taken from the videos. The authors suggested that their finding of stronger directionality for videos than pictures supports the processing-efficiency model (Beaumont, 1985; Mead and McLaughlin, 1992). According to this model, since an observer’s gaze seems biased to the right of an image, more material of a picture lies within the left visual field and is thus projected to the right hemisphere, which then allows more efficient processing of visual content and may thus provoke higher degree of pleasantness (Beaumont, 1985). When watching videos that show an object moving from left-to-right, such right-gaze bias, and as a consequence perception of the majority of the scenery by the right hemisphere, could become accentuated as compared to when watching pictures, resulting in more accentuated left-to-right bias in aesthetic judgments of videos than pictures (Friedrich et al., 2014). Furthermore, particular relevance of directionality for videos may also be predicted based on the processing fluency theory of aesthetic pleasure proposed by Reber et al. (2004). According to that theory, an observer’s socialization and experience play an important role in the formation of aesthetic preference. Consequently, for an observer who reads/writes from left-to-right and possesses corresponding visuomotor experience (Szego and Rutherford, 2008), actions evolving from left-to-right (i.e., in the direction of the observer’s familiar scanning direction) can be predicted to elicit stronger aesthetic pleasure (high fluency) than the same actions oriented right-to-left and thus against scanning direction (low fluency). Experiments reported by Topolinski (2010) provide direct support for that prediction as he found ocular-muscle training to provoke aesthetic preference in the trained direction.

Others’ actions unfolding in horizontal direction in front of an observer’s eyes are common in a variety of sports (Loffing et al., 2016), thus offering the possibility for directional bias to occur, for example, in performance evaluation (Maass et al., 2007; Kranjec et al., 2010). Maass et al. (2007) asked participants to watch videos of soccer goals and to rate the goals on a 9-point scale regarding strength, speed and beauty. Half of the videos showed goals evolving from left-to-right and right-to-left, respectively. In line with the above theoretical accounts, left-to-right readers (*n* = 72; Experiment 1) rated goals with a left-to-right trajectory as stronger ($\eta_{p}^{2}$ = 0.60), faster ($\eta_{p}^{2}$ = 0.62), and more beautiful ($\eta_{p}^{2}$ = 0.41) compared to goals with a right-to-left trajectory (a reversed effect was found in right-to-left readers; Experiment 3). Apart from soccer, directional bias in ratings could be even more relevant in sports where an athlete’s competition result depends, among others, on judges’ qualitative performance assessment. The latter is the case, for example, in the gymnastic discipline of balance beam where gymnasts perform from left-to-right and right-to-left in front of the judges sitting orthogonal to the beam. A gymnast’s final score is the sum of two separate scores – a difficulty (*D*)^1^ and an execution (*E*) score – minus potential penalties (e.g., due to timeout). The *E* score results from the assessment of performance, where judges begin at 10 points and reduce that score by a predefined value whenever they identify faults in an athlete’s execution of a skill. The assessment criteria used for deduction and formation of the *E* score relate to technical and artistic aspects of the performance, including their execution (e.g., posture), composition and aesthetics.^2^ In view of the evidence on directionality available so far, we expected that judgments related to the *E* score criteria in gymnastics (e.g., technique, posture and aesthetics) would turn out differently depending on a gymnast’s horizontal orientation. Specifically, in Experiment 2 (see section 4 below), we predicted that left-to-right readers would rate a gymnast’s performance shown in a left-to-right orientation higher compared to when the same performance is shown in a right-to-left orientation (cf. Maass et al., 2007).

The aforementioned hypotheses especially apply to people who are *not* used to or skilled in observing and evaluating others’ actions. As previous research on directionality did not include samples from populations that are specifically skilled for the experimental task at hand (e.g., see Maass et al., 2007; Kranjec et al., 2010), it is less clear whether directionality is an issue for people who *are* used to and skilled in observing and evaluating others’ actions such as sport judges. On the one hand, research indicates that skilled judges are characterized by task-specific perceptual-cognitive skills that allow them, for example, to accurately foresee events in a gymnast’s unfolding movement (e.g., Ste-Marie, 1999) as well as to make more accurate judgments in comparison to less or unskilled observers (e.g., Pizzera, 2015). Likewise, judges are experienced in watching and evaluating others’ movements performed from left-to-right and right-to-left. Consequently, fluency may occur irrespective of direction such that it could be suspected that skilled judges are less prone to bias induced by a gymnast’s horizontal orientation. On the other hand, despite of being highly trained in observing and evaluating others’ actions objectively, sport judges are known to not always perform optimally (Plessner and Haar, 2006; Hagemann et al., 2008; Mather, 2008). In artistic gymnastics, for example, judgment biases may result from viewing perspective (Plessner and Schallies, 2005), athletes’ reputation (Findlay and Ste-Marie, 2004) or the position of a gymnast in within-team order (Ansorge et al., 1978; Plessner, 1999). Furthermore, judges also have a favored reading/writing direction and it is reasonable to assume that one-directional experience in reading and writing is more comprehensive and intense compared to experience in observing and evaluating gymnasts’ actions. Likewise, judges are likely characterized by similar right hemisphere dominance for visuospatial processing, which is thought to promote a left-to-right bias (Levy, 1976; Beaumont, 1985; Mead and McLaughlin, 1992), as laypeople who took part in aforementioned research on directionality. Consequently, even skilled judges can be expected to demonstrate the directional bias suggested to occur in laypeople. We conducted the experiments reported below with this particular prediction in mind.

Here, in two experiments we sought to make a first step toward understanding the role of judging expertise in the occurrence of directional bias in perceptual judgments. To this end, we referred to artistic gymnastics and the discipline of balance beam in particular because judges are seated orthogonal to a gymnast’s direction of motion and see her moving from left-to-right and right-to-left. In Experiment 1, participants were presented with picture pairs showing a female gymnast performing a gymnastic or non-gymnastic action. Pairs were made up by vertically aligned content-wise identical images that only varied in horizontal orientation and participants were asked to choose the image from the pair which they found more beautiful. We expected that laypeople and judges would chose the picture showing an action oriented from left-to-right more often as beautiful than the picture showing the same action in right-to-left orientation (cf. Chokron and De Agostini, 2000; Ishii et al., 2011; Treiman and Allaith, 2013). In Experiment 2, participants were shown videos of gymnastic performances, with the same videos being shown in both left-to-right and right-to-left orientation across different trials. The participants’ task was to evaluate a gymnast’s performance on an 11-point scale according to the criteria aesthetics, technique, posture and overall. We expected that laypeople and judges would award more points to videos showing left-to-right as opposed to right-to-left oriented balance beam performances (cf. Maass et al., 2007).

## 2. General Method – Participants

We recruited a total of 96 left-to-right reading participants who were predominantly right-handed as the left-to-right directional bias is predicted to be most reliable in these individuals (Levy, 1976; McLaughlin et al., 1983; Karim et al., 2016). Forty-eight gymnastic judges [46 females; age: *M* = 24.79 years, *SD* = 10.72; judging experience in gymnastics: *M* = 7.38 years, *SD* = 6.27; judging licenses: 3 × A (highest), 2 × B, 21 × C, 22 × D] and 48 laypeople without experience in judging gymnastics or the like (25 females; age: *M* = 25.21 years, *SD* = 3.14) took voluntarily part in the two experiments. The majority of participants was right-handed according to a German version of Coren’s (1993) *Lateral Preference Inventory* (LPI; Büsch et al., 2009). One judge and three laypeople were classified as left-handers (LPI-score < 0), while one participant from the group of laypeople may be categorized as being mixed-handed (LPI-score = 0). All participants reported a left-to-right competence in reading and writing (judges: *M* = 18.77 years, *SD* = 10.73; laypeople: *M* = 18.88 years, *SD* = 3.26). No individual reported competence in reading or writing from right-to-left or top-to-down. One judge, however, reported competence in reading/writing from bottom-to-top for 22 years. Participants were naïve with regard to the purpose of the study and they provided written informed consent prior to the start of testing. All 96 participants took part in Experiments 1 and 2; the order of experiments was counterbalanced across participants.

In each group, the different test versions employed in Experiment 1 (2 versions; see section 3.2 Procedure for details) and Experiment 2 (12 versions; see section 4.2 Procedure for details) as well as the order of the two experiments (two orders) were fully combined. This procedure resulted in a total of 48 different combinations (see **Supplementary Material S1** for details). The sample size per group was realized based on that number in that each combination was filled with exactly one participant from the group of laypeople and judges, respectively. Furthermore, a priori sample size calculations with G^∗^Power 3.1.9.2 (Faul et al., 2007) showed that, to detect an expected effect of around Cohen’s *d* = 0.50 (cf. Nachson et al., 1999; Chokron and De Agostini, 2000; Friedrich et al., 2014) at α = 0.05 with 1-β = 0.80 using one-tailed one-sample or paired *t*-tests, a total sample size of *N* = 27 per group was required. To account for the potential spread in evidence underlying the above effect size, even if statistically significant (see Wetzels et al., 2011, for details), data were analyzed from both a classical and Bayesian perspective (see section 3.3 Data analysis below for details).

## 3. Experiment 1

This experiment aimed to verify that both laypeople and gymnastic judges would preferentially choose the picture with left-to-right oriented content when asked to select the more beautiful image from a picture pair (cf. Chokron and De Agostini, 2000; Ishii et al., 2011).

### 3.1 Apparatus and Stimuli

**FIGURE 1:**
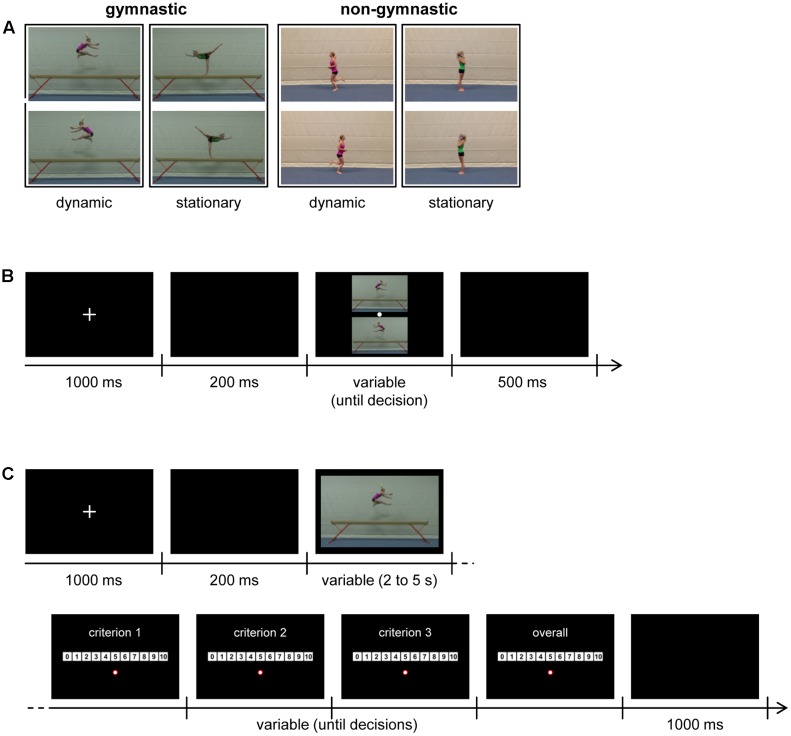
**(A)** Example picture pairs presented in Experiment 1: dynamic (wolf jump)/stationary (scale fwd.) gymnastic, dynamic (jogging)/stationary (phoning) non-gymnastic. Differences in color settings result from gymnastic and non-gymnastic stimuli being recorded with two different cameras. Importantly, this variation is irrelevant for the analysis of directionality in aesthetic preferences across all trials (“overall”). **(B)** Illustration of a trial in Experiment 1. **(C)** Illustration of a trial in Experiment 2. The order of criteria 1–3 (i.e., aesthetics, technique, posture) as well as the horizontal layout of the rating scale (i.e., 0 to 10 or 10 to 0) was counterbalanced across participants.

For the creation of experimental stimuli, a series of gymnastic and non-gymnastic action elements were recorded while performed individually by three female gymnasts. Different types of action elements were included since we wanted to additionally explore whether the hypothesized directionality in aesthetic preferences varies between action elements. Specifically, while no differences were expected for laypeople, we suspected that judges might demonstrate stronger aesthetic preference in non-gymnastic as opposed to gymnastic elements, because judges are used to observe and evaluate gymnastic as opposed to non-gymnastic actions. To enhance comparability between gymnastic and non-gymnastic elements, elements were performed by the same models in the same environment (i.e., gym) while wearing the same clothes (i.e., sports dress; for an illustration see **Figure 1A**).

#### Gymnastic Elements

Each gymnast performed dynamic and stationary gymnastic elements on a balance beam and their actions were recorded using a video camera (SONY HDR-FX1000e) that was positioned on a tripod orthogonal to the gymnast’s direction of motion (see **Figure 1A**). *Stationary* means that a gymnast put herself in a particular position (e.g., standing on one leg) and held that position for a moment. By contrast, an element was considered as *dynamic* when a gymnast moved horizontally and/or vertically without holding a particular position (e.g., jumping, turning, and rolling). Although pictures were presented in Experiment 1, video recordings of gymnastic elements were necessary because some elements selected for inclusion as pictures in this experiment were presented as videos in Experiment 2 (see 4.1 below for details; also, see Friedrich et al., 2014, for a similar approach). Each model performed gymnastic elements in both directions from the video camera’s perspective (i.e., from left to right and from right to left). Ten different dynamic and stationary elements were recorded for each model, thus totalling in 40 recordings per model (i.e., 10 dynamic/stationary elements × 2 directions) and 120 recordings across all three models. A full list of elements is provided in Table S1 of **Supplementary Material S1**.

Following the recordings, screenshots were taken from each video to obtain still images of an action (cf. Friedrich et al., 2014). With regard to *dynamic* elements, screenshots were taken from the “midpoint” of an action; for example, when an actor reached the maximum height in a jump element as illustrated in the left-most column of **Figure 1A**. With regard to *stationary* elements, screenshots were taken from the moment a gymnast achieved a stable position. For each dynamic and stationary element, one screenshot was then chosen from the pool of all recordings such that 10 pictures of different dynamic and 10 pictures of different stationary gymnastic elements were obtained.

There were three side conditions in the image selection process. First, to exclude that potential subtle differences in lighting could alternatively account for hypothesized lateral bias in aesthetic judgments, half of the selected elements showed a gymnast performing from left-to-right and right-to-left, respectively. Second, a gymnast should be positioned in/close to the center of an image while performing an element. Third, elements were chosen such that they were distributed almost equally across the three models.

#### Non-gymnastic Elements

Furthermore, for each of the above models pictures of 10 different dynamic and 10 different stationary *non*-gymnastic elements were taken with a hand-held mobile phone (Apple iPhone^®^ 5)^3^ from the same position from where video recordings of gymnastic elements were taken before. Each element was performed in both directions from the view of the camera (i.e., from left to right and from right to left), thus totalling again in 40 recordings per model (i.e., 10 dynamic/stationary × 2 directions) and 120 recordings across all three models. From the pool of recordings, 10 different dynamic and 10 different stationary non-gymnastic elements were selected using the same side conditions as in the selection process of gymnastic elements specified above.

#### Further Preparation

Following the selection of images depicting gymnastic and non-gymnastic elements, a horizontally mirrored version of each image was created using *Adobe Photoshop CS 4*. For each picture, the original and mirrored version was later used to form a vertically arranged picture pair in the experiment (see 3.2 Procedure below). That is, images of a pair were identical in content but varied only in orientation (i.e., left-to-right vs. right-to-left) as is illustrated in **Figure 1B**. Overall, 40 different picture pairs were presented as stimuli (dynamic/stationary × gymnastic/non-gymnastic; 10 picture pairs per combination). A complete overview of original and mirrored pictures included in the experiment is given in **Supplementary Material S2**. The experiment was programmed and run using the experimental software Experiment Builder (*SR Research*).

### 3.2 Procedure

Prior to the start of testing, participants received standardized written instructions as to the procedure and the experimental task. During testing, participants were seated and watched 40 different pairs of colored pictures in front of a black background, arranged on top of each other, on a 15.6" notebook monitor (Fujitsu Lifebook E754; monitor resolution set to 1920 pixel × 1080 pixel). Only one picture pair was shown per trial such that the experiment comprised 40 trials in total. In each picture pair, individual images were 880 pixel × 500 pixel in size (width × height), images were presented centrally in horizontal direction, and top/bottom images of picture pairs were the same distance apart from the screen’s center in vertical direction [i.e., 20 pixel vertical distance between the lower (upper) border of the top (bottom) image and the screen’s center].

Picture pairs were presented in random order and order was newly randomized for each participant. There were two different versions of the experiment: in version one (two), original images were always presented at the top (bottom) and horizontally mirrored images always at the bottom (top). Realization of the two versions was counterbalanced across participants in the group of judges and laypeople. Importantly, since half of the selected original images showed a model in left-to-right and right-to-left orientation, respectively, in both versions half of the top (bottom) images showed a model in left-to-right and right-to-left orientation. Control of vertical image position within pairs was considered necessary due to human’s tendency to associate good with up and bad with down (e.g., Casasanto, 2009).

In each trial, first a white fixation cross was presented in the center of a black screen for 1000 ms, followed by a blank black screen for 200 ms and then a picture pair until a decision was made by participants (**Figure 1B**). As in previous work (e.g., Nachson et al., 1999; Chokron and De Agostini, 2000; Ishii et al., 2011; Friedrich et al., 2014), the participants’ task was to indicate the picture they perceived as more beautiful (forced-choice decision). Participants responded by clicking on the more beautiful image (i.e., top or bottom) using a computer mouse. The mouse cursor was represented by a white dot 20 pixel × 20 pixel in size. To exclude a possible bias in the selection of an image due to mouse cursor position from a previous trial, in each trial the mouse cursor’s position was reset to the screen center and the mouse cursor was only visible on the screen where picture pairs were presented. After a choice was made, a blank black screen was shown for 500 ms before the next trial started. Completion of this experiment took about 8 min.

### 3.3 Data Analysis

For each participant, the proportion of selected images that showed an actor in left-to-right orientation was computed separately for the four action element conditions (10 trials per condition) and across all elements (“overall”; 40 trials). Mean proportions were tested against an expected proportion of 0.50 (random image selection) by means of one-sample *t*-tests separately for laypeople and judges. These tests were calculated one-tailed based on the directed hypothesis of an expected left-to-right bias; that is, that the proportion of “left-to-right”-image selection is larger than 0.50. The analytical software JASP (version 0.7.5.6) was used to calculate inferential statistics from both a classical and Bayesian perspective (JASP Team, 2016; see **Supplementary Material S1** for details). Estimation Software for Confidence Intervals (Cumming, 2012) was used to calculate an unbiased estimate of Cohen’s effect size *d*_unb_ and associated 95% confidence intervals.

With regard to the Bayesian perspective, we calculated the Bayes Factor (BF) as another measure of evidence (e.g., Kass and Raftery, 1995; Rouder et al., 2009; Wetzels et al., 2011). The BF is defined as the ratio of two conditional probabilities. Here we considered the ratio of the probability of the data under the hypothesis of a left-to-right bias (i.e., that the proportion of left-to-right picture selection would be above 50%) relative to the probability of the data under the hypothesis of no left-to-right bias (i.e., that the proportion of left-to-right picture selection would be equal to or lower than 50%). A BF value of 1 indicates evidence in favor of neither hypothesis. The more a BF value is above 1, the stronger the evidence in favor of the hypothesis of a left-to-right bias, whereas the more a BF value is smaller than 1, the stronger the evidence in favor of the hypothesis of no left-to-right bias. Apart from the raw BF values we also report evidence categories based on the output provided by JASP. Accordingly, for BF values larger than 1, categories are “anecdotal” (BF = 1–3), “moderate” (BF = 3–10), “strong” (BF = 10–30), “very strong” (BF = 30–100) and “extreme” (BF > 100), whereas for BF values smaller than 1, categories are “anecdotal” (BF = 1–1/3), “moderate” (BF = 1/3–1/10), “strong” (BF = 1/10–1/30), “very strong” (BF = 1/30–1/100) and “extreme” (BF < 1/100).

Raw data is available in **Supplementary Material S3**.

### 3.4 Results

**FIGURE 2:**
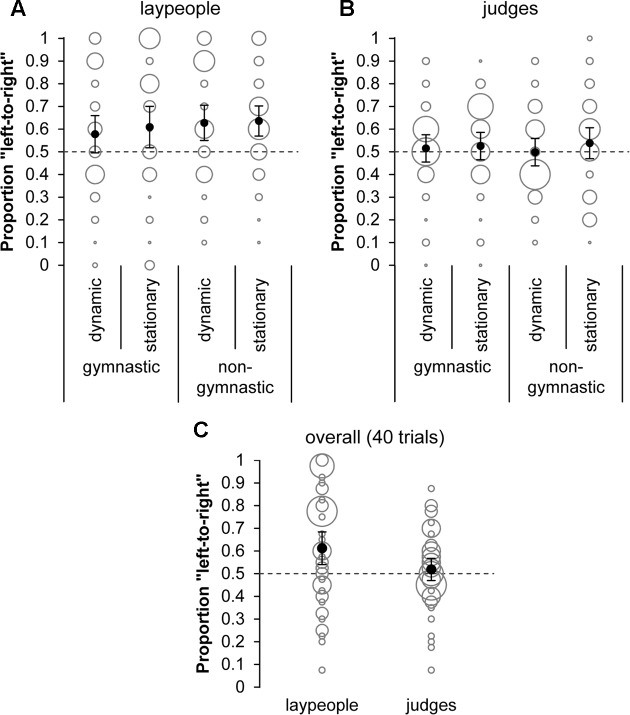
Proportion of pictures selected with a person’s left-to-right orientation relative to random selection (0.50; dotted line) separately for action element conditions in **(A)** laypeople and **(B)** judges and **(C)** overall (i.e., all trials) in laypeople and judges. Means are indicated by black dots and error bars illustrate two-sided 95% confidence intervals of the means. Gray bubbles represent single proportions with the size of bubbles indicating the frequency with which single proportions were observed.

**Figure 2** illustrates the results of aesthetic preference judgments observed relative to the proportion expected from random image selection and **Table 1** provides a summary of *t*-test results. In each action element condition (**Figure 2A**) and overall (**Figure 2C**), laypeople judged images showing a person oriented left-to-right as more beautiful than content-wise identical images showing a person in a right-to-left orientation. Gymnastic judges, however, did not demonstrate directionality for any action element (**Figure 2B**) nor overall (**Figure 2C**).

**Table 1:** Results from one-tailed one-sample *t*-tests in Experiment 1.

		Classical^a^				Bayesian^b^		
Group	Action element	*t*	*p*	*d*_unb_	95% CI	BF_+0_	Error %	Evidence
Judges	Dynamic gymnastic	0.487	0.314	0.069	(-0.213, 0.353)	0.239	~7.130e^-8^	Moderate_H0_
	Stationary gymnastic	0.833	0.204	0.118	(-0.164, 0.404)	0.344	~6.872e^-8^	Anecdotal_H0_
	Dynamic non-gymnastic	-0.069	0.528	-0.010	(-0.293, 0.273)	0.149	~7.204e^-8^	Moderate_H0_
	Stationary non-gymnastic	1.111	0.136	0.158	(-0.125, 0.444)	0.480	~6.412e^-8^	Anecdotal_H0_
	Overall (all elements)	0.770	0.223	0.109	(-0.173, 0.394)	0.320	~6.944e^-8^	Moderate_H0_
Laypeople	Dynamic gymnastic	1.892	0.032	0.269	(-0.017, 0.560)	1.559	~3.814e^-8^	Anecdotal^H+^
	Stationary gymnastic	2.390	0.010	0.339	(0.052, 0.634)	4.025	~1.929e^-8^	Moderate^H+^
	Dynamic non-gymnastic	3.286	<0.001	0.467	(0.173, 0.771)	32.127	~2.276e^-9^	Very strong^H+^
	Stationary non-gymnastic	4.109	<0.001	0.584	(0.283, 0.898)	306.195	~7.333e^-11^	Extreme^H+^
	Overall (all elements)	3.133	0.001	0.445	(0.153, 0.747)	21.887	~3.598e^-9^	Strong^H+^

*For all tests, the to-be-tested hypothesis was that the population mean is greater than 0.5. Therefore, all tests were calculated one-tailed. ^a^For classical *t*-tests *df* = 47. Effect size *d*_*unb*_ and associated 95% confidence intervals were calculated with the Estimation Software for Confidence Intervals (Cumming, 2012)*.

*^b^Bayesian *t*-tests were conducted using the statistical software JASP (version 0.7.5.6; JASP Team, 2016). A default Cauchy prior width of 0.707 was used. Strength of evidence was classified according to the evidence categories given in JASP. Note that in JASP, some evidence categories are labeled differently (i.e., “moderate” and “extreme”), for example, in comparison to the labels used by Wetzels et al. (2011) based on Jeffreys (1961) (i.e., “substantial” and “decisive”). Superscripts (H+) and subscripts (H0) denote strength of evidence toward the alternative and the null hypothesis, respectively. See **Supplementary Material S1** for results on Bayes factor robustness checks and sequential analyses of Bayes factors*.

Inspection of Bayes factors (Kass and Raftery, 1995; Rouder et al., 2009; Wetzels et al., 2011) indicates that laypeople data provide “anecdotal” (dynamic gymnastic) to “extreme” (stationary non-gymnastic) evidence in favor of the directed hypothesis of aesthetic preference for left-to-right oriented actions (see **Table 1**). When considered overall (i.e., all 40 trials without differentiation by action elements), data suggest “strong” evidence in favor of that hypothesis. Additional analyses indicate that evidence is robust for the “overall” condition (see Supplementary Figures S3A, S9A in the **Supplementary Material S1**). Conversely and as can already be anticipated from classical *t*-test-statistics, Bayes factors do not suggest evidence for the hypothesis of a left-to-right bias in aesthetic preferences in gymnastic judges. The Bayes factor associated with the overall condition rather indicates “moderate” evidence toward the null hypothesis of no “left-to-right”-image selection preference (i.e., left-to-right image selection frequency is equal to or lower than right-to-left image selection frequency). Bayes factor robustness checks (Supplementary Figures S2, S3B) and sequential analysis of Bayes factor development (Supplementary Figures S8, S9B) revealed that evidence was consistent and stable (albeit just in evidence categories anecdotal to moderate) against the hypothesis of a preference for left-to-right oriented actions (see **Supplementary Material S1** for details).

An initial 2 (Group) × 2 (Type: gymnastic vs. non-gymnastic) × 2 (Action: stationary vs. dynamic) mixed ANOVA with repeated measures on the last two factors did not reveal a statistically significant main effect for Type (*p* = 0.235) or Action (*p* = 0.127) nor meaningful interactions (*p* = 0.186 for Group × Type; *p*s > 0.250 otherwise). Therefore, data were collapsed across conditions before examining group differences in aesthetic preferences. A one-tailed *t*-test for independent samples on “overall” left-to-right image selection frequencies was considered justified as judges should have reduced bias (cf. section 1 Introduction) as compared to laypeople. Accordingly, laypeople had stronger “left-to-right”-image preference than judges, *t*(82.93) = 2.155, *p*_one-tailed_ = 0.017, *d*_unb_ = 0.44, 95% CI [0.034, 0.844]. The Bayes factor associated with this comparison, however, suggests that the data provide almost “anecdotal” evidence in favor of the non-directional hypothesis of group differences in “left-to-right”-preferences, BF_10_ = 3.199, error % = 6.584e^-5^ (for details see Supplementary Figures S4, S10 in the **Supplementary Material S1**).

Additional analyses revealed similar bias in male and female laypeople’s aesthetic preference and no meaningful relationship between participants’ age (laypeople or judges) or years of judging experience and “left-to-right”-image selection frequency (see **Supplementary Material S1** for details). Moreover, inspection of top image selection frequencies highlights the importance of controlling the vertical position of images within pairs – as was done in the experiment – since both laypeople and judges tended to preferentially perceive the top image as more beautiful.

In summary, findings from this experiment are congruent with previously reported left-to-right bias in aesthetic preference (Chokron and De Agostini, 2000; Ishii et al., 2011; Treiman and Allaith, 2013; Friedrich et al., 2014). That bias, however, was only evident in laypeople but not in gymnastic judges.

## 4. Experiment 2

In this experiment, we aimed to verify that both laypeople and gymnastic judges would rate gymnastic performances shown in left-to-right orientation higher compared to the same performances shown in right-to-left orientation (cf. Maass et al., 2007). Participants were identical to Experiment 1.

### 4.1 Apparatus and Stimuli

The 10 dynamic and a selection of six stationary gymnastic elements shown as pictures in Experiment 1 were presented as videos in this experiment (see **Supplementary Table S1** in the **Supplementary Material S1** for details; non-gymnastic actions were not included in this experiment). Each element was shown in original and mirrored orientation. Our prime interest was in the participants’ evaluation of videos showing dynamic gymnastic elements in left-to-right vs. right-to-left orientation. In these elements, a gymnast changes position in horizontal and/or vertical direction and these directional changes were assumed to most reliably elicit directionality in participants’ evaluations (see Maass et al., 2007; Friedrich et al., 2014). Videos of stationary gymnastic elements were included as a sort of “fillers” to allow participants to familiarize with evaluating gymnastic performances (see also 4.2 Procedure below) and to enhance variation in stimulus material.

Videos were edited using *Adobe Premiere Pro CS 4* such that the frame showing a picture from Experiment 1 was in the middle of a video (cf. Friedrich et al., 2014). Half of the videos showed actions that were originally performed from left-to-right and right-to-left, respectively. For each video, horizontally mirrored versions were created such that two content-wise identical versions of an action were available that differed in terms of the horizontal direction of an action only (i.e., left-to-right vs. right-to-left). Videos lasted between 2 and 5 s, video size was 1280 pixel × 720 pixel (width × height) and the frame rate was 25 frames per second. Importantly, the mirroring technique ensured that video specifications were identical for original and mirrored versions, the only difference being the horizontal orientation of video content. Collectively, a total of 32 videos were included as stimuli. Experimental software Experiment Builder (*SR Research*) was used to design and run the experiment.

### 4.2 Procedure

Prior to the start of the experiment, participants received standardized written instructions with regard to the procedure and the experimental task. Instructions included brief explanations of the four performance criteria to ensure that participants were aware of what was meant with technique, posture, aesthetics and overall assessment. Participants were allowed further inquiry with regard to instructions and these were addressed by the experimenter as long as inquiries were not related to the experiment’s underlying research question or hypothesis. During testing, participants were seated and watched colored videos in front of a black background on a 15.6" notebook monitor (Fujitsu Lifebook E754; monitor resolution set to 1280 pixels × 720 pixels). Videos were centered horizontally and vertically on the screen.

Videos were presented in two consecutive blocks including either original or horizontally mirrored clips only and the order of blocks was randomized across participants. Blocked presentation of original and mirrored clips was considered necessary to exclude the possibility that the same element was presented in different orientation in consecutive trials. Otherwise, in the case of such event participants might have recognized that the second version was identical to the first except for mirroring and consequently might have rated the second version the same as the first based on memorized ratings for the first version. Moreover, within each block, videos of the six stationary elements were presented first before the 10 videos of dynamic elements were shown. We decided to do so to allow participants some trials of familiarization with evaluating the gymnasts’ actions before being confronted with dynamic elements which we were primarily interested in. Within the respective sub-blocks of stationary and dynamic elements, the order of videos was newly randomized for each participant. Importantly, within blocks and sub-blocks, half of the videos showed actions from left-to-right and right-to-left, respectively.

In each trial (see **Figure 1C**), first a white fixation cross was presented in the center of a black screen for 1000 ms, followed by a blank black screen for 200 ms and then a video of a gymnastic element. Upon the end of a video, in four consecutively presented response screens participants were asked to rate the previous action according to the criteria technique, posture, aesthetics and overall assessment. These criteria were chosen in dependence on the E-panel’s task in gymnastic judging, which is the evaluation of performance to determine an execution score^4^. While the overall assessment was always required as final evaluation, the order of the three other criteria was fully varied and counterbalanced across, but kept constant within participants. Performance evaluations were made on a 11-point scale ranging from 0 (absolutely inadequate) to 10 (absolutely outstanding). In each evaluation step, the required criterion was written on top of the screen centered horizontally. Ratings were made by clicking with a mouse cursor (represented by a red framed white dot that was 20 pixels × 20 pixels in size) on a numeric scale ranging from 0 to 10. On each rating screen, the scale was always centered horizontally and vertically and the mouse cursor position appeared at a constant position (horizontally centered below the rating scale; coordinates: *x* = 640 pixels, *y* = 500 pixels from the top of the monitor). The mouse cursor was only shown on the four rating screens and not visible otherwise during a trial. Two different types of rating scales were employed – i.e., ranging either from 0 to 10 or from 10 to 0 (left-to-right) – to control for a possible spatial bias in participants’ ratings (Maass et al., 2007). Different rating scales were counterbalanced across participants. The full combination of different rating scales with different orders of the first three evaluation criteria resulted in 12 different test versions for this experiment. After the overall assessment was made on the fourth rating screen, a blank black screen was shown for 1000 ms before the next trial started. Completion of this experiment took about 15 min.

### 4.3 Data Analysis

For each participant, we calculated the mean rating scores for dynamic gymnastic elements separately for action direction (i.e., 10 trials left-to-right and 10 trials right-to-left) for each performance criterion. Initial inspection of dependent measures indicated high positive correlations between measures within and across groups of participants (*r*s ≥ 0.786, *p*s < 0.001). For each criterion, we compared mean ratings for actions oriented left-to-right and right-to-left separately in judges and laypeople by means of *t*-tests for paired samples. Tests were calculated one-tailed based on the hypothesis of a left-to-right directional bias in participants’ performance evaluation (Maass et al., 2007). JASP (version 0.7.5.6) was used to obtain statistics from both a classical and Bayesian perspective (JASP Team, 2016) and ESCI was used to calculate 95% CIs for effect size *d*_unb_ (Cumming, 2012). Raw data is also available in **Supplementary Material S3**.

### 4.4 Results

**FIGURE 3:**
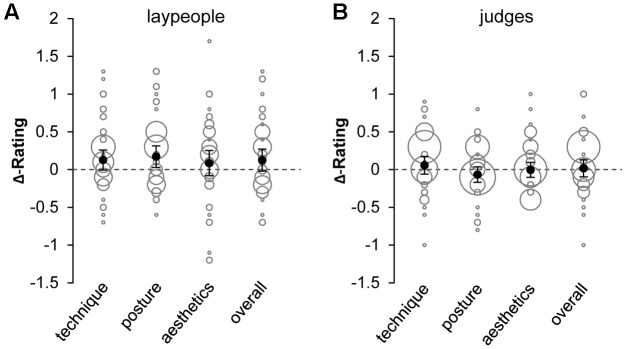
Differences in performance ratings of dynamic gymnastic elements in left-to-right vs. right-to-left orientation for each evaluation criterion in **(A)** laypeople and **(B)** judges. Positive (negative) values indicate better (worse) evaluation of left-to-right elements. Black dots indicate mean differences and error bars illustrate two-sided 95% confidence intervals of the means. Gray bubbles represent single differences with the size of bubbles indicating the frequency with which single differences were observed.

**Figure 3** illustrates mean differences in ratings for left-to-right vs. right-to-left dynamic gymnastic actions on each criterion in laypeople and judges, respectively. A positive (negative) rating difference indicates better (worse) evaluation of left-to-right compared to right-to-left actions. Descriptively, there was a slight, consistent trend of better evaluation of left-to-right oriented actions in laypeople (**Figure 3A**), but not in gymnastic judges (**Figure 3B**). Results obtained from one-tailed *t*-tests provide very tentative indication of a directional bias in laypeople’s performance evaluation (see **Table 2**). However, mean differences and effect sizes *d*_unb_ were clearly small and the ranges of 95% CIs associated with effect sizes do not strengthen reliability of the effects. Except for the criterion “aesthetics”, Bayes factors indicate only “anecdotal” to low “moderate” evidence toward the hypothesis of better evaluation of left-to-right than right-to-left actions in laypeople. Additional Bayes factor robustness checks (Supplementary Figure S5) and sequential analyses of Bayes factor development (Supplementary Figure S11) call for a cautious interpretation of a potential left-to-right bias in laypeople’s evaluations. In judges, Bayes factors suggest “anecdotal” to “strong” evidence toward the null hypothesis (i.e., no effect of horizontal motion direction on performance evaluation or better evaluation of right-to-left than left-to-right oriented actions). Findings from Bayes factor robustness checks (Supplementary Figure S6) and sequential analyses of Bayes factor development (Supplementary Figure S12) indicate that the evidence is quite stable (see **Supplementary Material S1** for details).

**Table 2:** Results from one-tailed paired *t*-tests in Experiment 2 (dynamic gymnastic elements only).

Group	Criterion	Classical^a^				Bayesian^b^		
		*t*	*p*	*d*_unb_	95% CI	BF_+0_	Error %	Evidence
Judges	Technique	0.932	0.178	0.049	(-0.056, 0.156)	0.385	~6.737e^-8^	Anecdotal_H0_
	Posture	-1.356	0.909	-0.059	(-0.147, 0.028)	0.071	~5.785e^-8^	Strong_H0_
	Aesthetics	-0.127	0.550	-0.006	(-0.093, 0.081)	0.143	~7.204e^-8^	Moderate_H0_
	Overall assessment	0.262	0.397	0.013	(-0.087, 0.114)	0.194	~7.188e^-8^	Moderate_H0_
Laypeople	Technique	1.896	0.032	0.121	(-0.007, 0.252)	1.569	~3.799e^-8^	Anecdotal^H+^
	Posture	2.384	0.011	0.157	(0.024, 0.294)	3.978	~1.947e^-8^	Moderate^H+^
	Aesthetics	1.001	0.161	0.072	(-0.072, 0.218)	0.418	~6.630e^-8^	Anecdotal_H0_
	Overall assessment	1.696	0.048	0.117	(-0.021, 0.257)	1.121	~4.593e^-8^	Anecdotal^H+^

*For all tests, the to-be-tested hypothesis was that actions evolving from left-to-right are evaluated better than actions from right-to-left. Accordingly, *p*-values are one-tailed. ^a^For classical *t*-tests *df* = 47. Effect size *d*_*unb*_ and associated 95% confidence intervals were calculated with the Estimation Software for Confidence Intervals (Cumming, 2012)*.

*^b^Bayesian *t*-tests were conducted using the statistical software JASP (version 0.7.5.6; JASP Team, 2016). A default Cauchy prior width of 0.707 was used. Strength of evidence was classified according to the evidence categories given in JASP. Note that in JASP, some evidence categories are labeled differently (i.e., “moderate” and “extreme”), for example, in comparison to the labels used by Wetzels et al. (2011) based on Jeffreys (1961) (i.e., “substantial” and “decisive”). Superscripts (H+) and subscripts (H0) denote strength of evidence toward the alternative and the null hypothesis, respectively. See **Supplementary Material S1** for results on Bayes factor robustness checks and sequential analyses of Bayes factors*.

Additional analyses (see **Supplementary Material S1** for details) revealed that, on average, laypeople awarded higher ratings than judges (range: $\eta_{p}^{2}$ = 0.080–0.189) and that female laypeople awarded higher ratings than male laypeople (range: $\eta_{p}^{2}$ = 0.087–0.137). There was no indication of a reliable relationship between participants’ age (calculated separately for laypeople and judges) or years of judging experience and rating differences (left-to-right minus right-to-left) for any performance criterion. Also, across experiments, there was no meaningful association between directionality in aesthetic preference (Experiment 1) and performance evaluation (Experiment 2) neither in laypeople (correlations, however, were consistently positive; range: *r* = 0.175–0.251) nor in judges. Finally, additional inclusion of evaluations of stationary gymnastic elements resulted in an even smaller directionality effect especially in laypeople. This was not unexpected because stationary elements lack an actor’s positional change and were therefore assumed to be less likely to induce directionality in performance evaluation.

## 5. Discussion

Left-to-right readers were reported to demonstrate a left-to-right directional bias in aesthetic preferences (e.g., Chokron and De Agostini, 2000; Ishii et al., 2011; Friedrich et al., 2014) and performance evaluation (Maass et al., 2007). Here we tested the hypothesis that such bias is evident in left-to-right reading laypeople and gymnastic judges when selecting the more beautiful image from a vertically aligned picture pair (Experiment 1) and when evaluating gymnasts’ balance beam performances (Experiment 2).

Data from Experiment 1 is in favor of the left-to-right bias in aesthetic preference. However, only laypeople, but not gymnastic judges, demonstrated the bias as indicated by tests against random image selection and group comparisons. Directionality in laypeople is in line with previous research on unskilled observers (Chokron and De Agostini, 2000; Ishii et al., 2011; Treiman and Allaith, 2013; Friedrich et al., 2014), suggesting that left-to-right reading habit is associated with left-to-right bias in aesthetic judgments.

Reasons for the absence of directionality in judges’ aesthetic judgments are difficult to pinpoint in view of the experiment’s underlying design. Consequently, in the following we can only speculate about possible mechanisms and, by doing so, stimulate directions for future research. First, non-directionality could stem from judges’ experience in observing and evaluating actions evolving from both left-to-right and right-to-left, resulting in direction-independent fluency and consequently no directional preference in aesthetic judgments. This would fit with findings of attenuated or no directional bias in bilingual individuals who read and write in both directions (e.g., Morikawa and McBeath, 1992; Maass and Russo, 2003; Szego and Rutherford, 2008). However, judging in gymnastics does not involve exclusive observation of actions in one particular direction (e.g., right-to-left) so that judges’ reading/writing habit cannot be counteracted systematically as in aforementioned bilinguals. A bias-attenuating effect of judging experience seems remarkable insofar as our judges had, on average, about 11 years longer experience in reading and writing exclusively from left-to-right compared to judging in gymnastics. To test the attenuating effect more directly, in future experiments subjects’ experience in observing and evaluating others’ actions could be systematically varied before asking them to make aesthetic judgments (cf. Topolinski, 2010).

Second, the absence of judges’ directionality could additionally be due to their processing strategy. The fact that our study was mostly about gymnastics (i.e., a sport that judges are highly attracted to) and the presentation of gym-specific stimuli (i.e., all pictures were recorded in a gym and all actions were performed by the same females wearing sports clothes for both gymnastic and non-gymnastic actions) might have induced high motivation and personal relevance of the experimental task in judges. This, in turn, may have led to a more systematic or analytic processing strategy (e.g., Bohner et al., 1995; Reber et al., 2004), resulting in the tendency to be less biased by picture orientation (for an analogy in the occurrence of the framing effect, see McElroy and Seta, 2003). Laypeople, in contrast, may have primarily adopted a heuristic strategy, for example, due to lower personal relevance of the task, thus facilitating directional bias to occur. One way to address this further could be to manipulate participants’ personal relevance of an aesthetic judgment task (cf. McElroy and Seta, 2003). To the best of our knowledge, the role of motivation, task engagement or relevance has not been considered in research on directionality in aesthetic judgments, but doing so could turn out fruitful to better understand the (complexity of the) mechanisms that are potentially involved in directional bias apart from fluency.

Data from Experiment 2 indicate that laypeople, but not judges, might tend to evaluate left-to-right oriented dynamic gymnastic elements better than identical elements oriented from right-to-left. While this would partly fit with previous reports (Maass et al., 2007) and our initial predictions, it clearly is a very tentative conclusion because neither effect sizes and associated 95% confidence intervals nor Bayes factors provide convincing evidence in favor of the hypothesis of left-to-right directional bias in the evaluation of dynamic performances. The trend of directionality even disappears when evaluations of stationary elements are included in the analyses (see **Supplementary Material S1**).

The absence of a distinct left-to-right bias, especially in laypeople, was unexpected as previously reported effects associated with such bias in left-to-right readers were large (see Maass et al., 2007). Likewise, based on the findings of Friedrich et al. (2014) and the predictions derived from both the processing-efficacy model (Beaumont, 1985; Mead and McLaughlin, 1992) and the processing fluency theory (Reber et al., 2004; see also Topolinski, 2010), we expected that the bias should be identified for performance evaluation in our experiment. We speculate that, apart from differences in stimuli, task instructions or demands may have triggered different processing strategies that could explain (part of the) differences between study findings as well. Maass et al. (2007) instructed participants to rate videos on a 9-point scale regarding three different variables (strength: “How strongly does the player hit the ball?”, speed: “How fast is the goal?”, beauty: “How beautiful is the goal?”), whereas Friedrich et al. (2014) presented pairs of videos and asked participants to select the video they found aesthetically more pleasant. Similar to our explanation for the occurrence of a left-to-right bias in laypeople’s aesthetic judgments in Experiment 1 (see above; cf. Bohner et al., 1995; McElroy and Seta, 2003; Reber et al., 2004), instructions in the previous studies might have induced a rather heuristic or holistic strategy in participants, thereby facilitating a left-to-right bias. By contrast, in our Experiment 2, participants were implicitly required to specifically monitor and attend to a gymnast’s movement for proper performance evaluation. Consequently, task demands may have made participants preferentially adopt a more systematic or analytic strategy, leading to more conscious control processes (cf. Lleras and Von Mühlenen, 2004; Smilek et al., 2006, for related discussions) and this could have prevented the occurrence of clear directional bias. Such a scenario could be particularly relevant to explain the weak directionality in the group of laypeople. As discussed above for Experiment 1, and not mutually exclusive to the aforementioned explanation, absence of bias in the group of judges may have additionally resulted from their experience in observing and evaluating gymnasts’ actions. We acknowledge that the exact mechanisms underlying the null finding are difficult to pinpoint. On a more positive note, however, new questions arise, for example, concerning the role of task instructions or demands for the occurrence of directional bias and related processes (e.g., see Smilek et al., 2006; Watson et al., 2010). In this regard, our research hopefully encourages an extension and more comprehensive discussion of the factors and psychological mechanisms that potentially contribute to and underlie directionality, respectively.

In summary, the two experiments reported here indicate that, as a group, judges are less prone to directional bias than laypeople. Consequently, despite some inter-individual variation being apparent in both groups in Experiments 1 and 2 (see **Figures 2**, **3**), findings preliminarily suggest that directionality may rather be an issue for *un*skilled but not for *skilled* judging. The mechanisms underlying a potential skill effect, however, still need to be ruled out. To further unravel the role of an actor’s direction on perceptual judgments and to broaden theoretical perspectives in this endeavor, future work may consider the directions outlined above as well as take into account the manipulation of variables that are known to reduce judges’ cognitive capacity and enhance their proneness to bias (e.g., time pressure, task complexity; Damisch and Mussweiler, 2009).

## Ethics Statement

The study was carried out in accordance with the recommendations of the local ethics committee at the Department of Social Sciences at the University of Kassel with written informed consent from all subjects. All subjects gave written informed consent in accordance with the Declaration of Helsinki. The protocol was approved by the ethics committee at the Department of Social Sciences at the University of Kassel (code E05201503).

## Author Contributions

FL and SN developed the study concept. All authors contributed to the study design. Testing and data collection were performed by SN. FL performed the data analyses. All authors contributed substantially to the interpretation of data. FL drafted the manuscript, SN and NH provided critical revisions for important intellectual content. All authors approved the final version of the manuscript for submission. All authors agree to be accountable for all aspects of the work.

## Conflict of Interest Statement

The authors declare that the research was conducted in the absence of any commercial or financial relationships that could be construed as a potential conflict of interest.

## Footnotes

1 In artistic gymnastics, the *D* score is defined by the eight (women) or 10 (men) most difficult elements performed, with each element being associated with a predefined difficulty value.

2 Rules in artistic gymnastics for both women’s and men’s competition can be accessed via http://www.fig-gymnastics.com/site/rules/disciplines/art.

3 Initial inspection of video camera recordings of dynamic non-gymnastic elements indicated that some recordings could not be used because these were blurred due to the gymnasts’ motion. We therefore used an Apple iPhone^®^ 5 to capture non-gymnastic elements.

4 http://www.fig-gymnastics.com

## Supplementary Material

SUPPLEMENTARY MATERIAL S1 Supplementary details on stimulus material, determination of sample size and additional analyses.

SUPPLEMENTARY MATERIAL S2 Images presented in Experiment 1.

SUPPLEMENTARY MATERIAL S3 Combined raw dataset for Experiments 1 and 2.
