# Supplementary material for: Directionality in Aesthetic Judgments and Performance Evaluation: Sport Judges and Laypeople Compared
Source: Front Psychol. 2017 Dec 5;8:2109. doi: 10.3389/fpsyg.2017.02109 (PMC5723409; doi:10.3389/fpsyg.2017.02109)
Supplement: SUPPLEMENTARY MATERIAL S1 — Supplementary details on stimulus material, determination of sample size and additional analyses. [file Supplementary_Material_S1.PDF]

Directionality in aesthetic judgments and performance evaluation: Sport judges and laypeople compared

Content

Stimulus Material 2

Determination of Sample Size 3

Additional Analyses 4

Does participants' sex moderate directionality? 4

Age and years of judging experience 4

Top image selection frequencies (Exp. 1) 5

Comparison of laypeople's and judges' evaluations of dynamic gymnastic actions (Exp. 2) ... 6

Performance evaluation in all trials (dynamic & stationary gymnastic elements; Exp. 2) 7

Association between directionality in individuals' aesthetic preference (Exp. 1) and performance evaluation (Exp. 2) 8

Bayes factors robustness checks 8

Sequential analysis of Bayes factors (with robustness check) 14

Stimulus Material

Table S1. Stationary and dynamic gymnastic and non-gymnastic action elements shown as pictures in Exp. 1. Stationary gymnastic elements set in italics were also shown as videos in Exp. 2 (dynamic gymnastic elements were also shown in Exp. 2).

gymnastic				*non-gymnastic*			
stationary		*dynamic*		*stationary*		*dynamic*	
element	original orientation	element	original orientation	element	original orientation	element	original orientation
v-sit (with support of hands behind the back)	L-to-R	scissors leap fwd.	L-to-R	reading a book	L-to-R	kicking	L-to-R
front lying support (arms stretched)	L-to-R	roll fwd.	L-to-R	looking through field glasses	L-to-R	inline skating	L-to-R
pose (sitting; outer leg stretched towards the ground, other leg bent and foot contacts knee of other leg; arms stretched left/right)	L-to-R	stretched jump (only as picture in Exp. 1, not as video in Exp. 2)	L-to-R	browsing through a photo album	L-to-R	bowling	L-to-R
pose (knee stand)	L-to-R	stretched jump with single leg change	L-to-R	relaxing in a deck chair	L-to-R	brushing	L-to-R
straddle support	L-to-R	step-close steps fwd.	L-to-R	raising a trophy	L-to-R	rope skipping	L-to-R
knee arabesque	R-to-L	tuck jump	R-to-L	drinking from a water bottle	R-to-L	playing badminton	R-to-L
pose (standing; one leg in front in tiptoe posture; both arms stretched and pointing up)	R-to-L	cat leap	R-to-L	working on a note-book	R-to-L	riding a scooter	R-to-L
scale fwd.	R-to-L	wolf jump	R-to-L	doing push-up	R-to-L	jogging	R-to-L
v-sit (free; i.e., without support of the hands)	R-to-L	split leap fwd.	R-to-L	putting on eye make-up	R-to-L	pulling a bag	R-to-L
handstand	R-to-L	turn on one foot (360°)	R-to-L	phoning	R-to-L	skateboarding	R-to-L
		stretched jump with double leg change (not as picture in Exp. 1, only as video in Exp. 2)	L-to-R				

Note: Entries in the “original orientation” columns indicate whether an action element was originally performed from left-to-right (L-to-R) or from right-to-left (R-to-L) from the recording camera’s perspective.

Determination of Sample Size

Sample size for each group resulted from the full combination of different test versions employed in Experiments 1 and 2. In Exp. 1, we created 2 versions differing in the vertical positioning of the two images of a picture pair. In Exp. 2, we created 6 versions differing in the order of the first three evaluation criteria (i.e., aesthetics, technique and posture) and these versions were run either with an evaluation scale ranging from 0 to 10 or from 10 to 0 (arranged from left-to-right) to control for potential spatial bias in judgments. Thus, Exp. 2 comprised 12 different versions. Full combination of the versions used in Exp. 1 and Exp. 2 (i.e., 2 x 12 = 24 versions) and counterbalancing the order of the two experiments resulted in a total of 48 different test arrangements. Inclusion of more than one participant per test arrangement was difficult to achieve particularly for the group of gymnastic judges. Consequently, prior to the start of testing we decided to test 48 participants in each group of judges and laypeople.

Moreover, based on an initial inspection of previously identified effects in directionality research (e.g., Chokron & De Agostini, 2000; Friedrich, Harms, & Elias, 2014; Ishii, Okubo, Nicholls, & Imai, 2011), when planning the study we expected to unravel effects around Cohen's $d = 0.50$. A priori sample size calculations run with G*Power 3.1.9.2 (Faul, Erdfelder, Lang, & Buchner, 2007) revealed that, to detect such an effect at $\alpha = 0.05$ with $1-\beta = 0.80$ using one-tailed one-sample or paired *t*-tests (owing to our directed hypotheses of expected left-to-right bias in aesthetic preference and performance evaluation), required a total sample size of only $N = 27$ per group (for completeness: $N = 34$ for two-tailed tests).

Additional Analyses

Does participants' sex moderate directionality?

Males were recently suggested to demonstrate a stronger left-to-right directional bias in aesthetic preference than females (Friedrich et al., 2014). Consequently, the fact that in our study the majority of judges were female (i.e., 46 out of 48 individuals) could alternatively explain the absence of directional bias that group. We checked whether the basic assumption (i.e., that females would be less prone to left-to-right directionality than males) actually holds in our data. To this end, we reanalysed male and female laypeople's performance.

Experiment 1. The proportion of left-to-right image selection was similar in males (M = 0.615, SD = 0.229) and females across all trials (M = 0.609, SD = 0.268), $t(46) = 0.086$, $p = .932$. When tested against above random image selection (i.e., 0.50), directionality was slightly more pronounced in males, $t(22)= 2.413$, $p_{\text{one-tailed}} = .012$, $d_{\text{unb}} = 0.486$, 95% CI [0.063, 0.933], than females, $t(24)= 2.031$, $p_{\text{one-tailed}} = .027$, $d_{\text{unb}} = 0.393$, 95% CI [-0.006, 0.810].

Experiment 2. Females awarded better ratings than males on each evaluation criterion (all ps < .05; technique: $\eta_p^2 = .087$; posture: $\eta_p^2 = .102$; aesthetics: $\eta_p^2 = .137$; overall: $\eta_p^2 = .127$), but two-factorial mixed ANOVAs did not indicate an interaction between participants' sex and action orientation.

Collectively, in contrast to the findings by Friedrich et al. (2014) and in line with the data reported by Maass, Pagani, and Berta (2007), our laypeople data does not indicate that participants' sex moderates directionality.

Age and years of judging experience

Experiment 1. Further exploration of data at an individual level did not indicate a relationship between the participants' age and "left-to-right"-image selection neither in judges, $r = -.102$, $p = .490$, $BF_{10} = 0.227$, nor in laypeople, $r = .002$, $p = .992$, $BF_{10} = 0.180$ (beta* prior width = 1 for calculations of BF_{10}). Also, there was no relationship between the years of judging experience and the overall proportion of "left-to-right"-image selection in gymnastic judges, $r = -.069$, $p = .643$, $BF_{10} = 0.200$.

Experiment 2. At an individual level, there was no indication of a linear relationship between the participants' age and rating differences for any performance criterion neither in judges (technique: $r = .111$, $p = .454$, $BF_{10} = 0.236$; posture: $r = .109$, $p = .461$, $BF_{10} = 0.234$; aesthetics: $r = -.218$, $p = .137$, $BF_{10} = 0.527$; overall: $r = .036$, $p = .810$, $BF_{10} = 0.185$) nor in laypeople (technique: $r = .056$, $p = .707$, $BF_{10} = 0.193$; posture: $r = .011$, $p = .940$, $BF_{10} = 0.180$; aesthetics: $r = -.007$, $p = .960$, $BF_{10} = 0.180$; overall: $r = -.040$, $p = .790$, $BF_{10} = 0.186$). Likewise, in the group of gymnastic judges, there was no indication of an association between years of judging experience and rating differences (technique: $r = .146$, $p = .323$, $BF_{10} = 0.289$; posture: $r = .073$, $p = .622$, $BF_{10} = 0.202$; aesthetics: $r = -.195$, $p = .183$, $BF_{10} = 0.425$; overall: $r = .055$, $p = .709$, $BF_{10} = 0.193$).

Top image selection frequencies (Exp. 1)

Previous work suggests that positive attributes are more likely associated with top (or up) than bottom (or down) (e.g., Casasanto, 2009, Exp. 1). Therefore, we controlled the vertical position of an image within a picture pair such that, across picture pairs presented to participants, half of the top images showed an action oriented left-to-right and right-to-left, respectively.

Here we tested whether participants were actually inclined to more often perceive the top image of a picture pair as more beautiful. To this end, for each participant the proportion of top image selection was calculated for each of the four action element conditions (i.e., dynamic/stationary x gymnastic/non-gymnastic) and overall. Mean proportions were then tested against random image selection (i.e., 0.5) separately within laypeople and judges. Descriptive data and inferential statistics are summarized in Table S2 below. As is clear from the data, participants indeed tended to perceive the top image of a picture pair as more beautiful. This highlights the necessity to control vertical image position when presenting picture pairs as was done in our experiment.

Table S2. Summary statistics on top image selection frequencies.

			Classical[a]				Bayesian[b]	
group	action element	*M (SD)*	*t*	*p*	d_{unb}	*95% CI*	BF_{10}	error %
judges	dynamic gymnastic	0.556 (0.171)	2.276	.027	0.323	(0.036, 0.617)	1.624	$2.319e^{-8}$
	stationary gymnastic	0.579 (0.210)	2.608	.012	0.370	(0.081, 0.667)	3.223	$1.288e^{-8}$
	dynamic non-gymnastic	0.548 (0.192)	1.725	.091	0.245	(-0.040, 0.535)	0.619	$4.480e^{-8}$
	stationary non-gymnastic	0.571 (0.173)	2.844	.007	0.404	(0.114, 0.704)	5.482	$7.668e^{-9}$
	overall (all elements)	0.564 (0.126)	3.491	.001	0.496	(0.201, 0.802)	27.436	$1.143e^{-9}$
laypeople	dynamic gymnastic	0.544 (0.177)	1.709	.094	0.243	(-0.042, 0.533)	0.603	$4.553e^{-8}$
	stationary gymnastic	0.567 (0.159)	2.907	.006	0.413	(0.123, 0.713)	6.343	$6.588e^{-9}$
	dynamic non-gymnastic	0.569 (0.126)	3.788	< .001	0.538	(0.241, 0.848)	61.485	$3.555e^{-10}$
	stationary non-gymnastic	0.606 (0.160)	4.591	< .001	0.652	(0.347, 0.973)	641.581	$9.796e^{-13}$
	overall (all elements)	0.571 (0.109)	4.554	< .001	0.646	(0.342, 0.966)	573.547	$1.788e^{-12}$

Note: All tests were calculated two-tailed.

[a] For classical t-tests $df = 47$. Effect size d_{unb} and associated 95% confidence intervals were calculated with the Estimation Software for Confidence Intervals (Cumming, 2012).

[b] Bayesian t-tests were conducted using the statistical software JASP (version 0.7.5.6; JASP Team, 2016) with a default Cauchy prior width of 0.707.

Comparison of laypeople's and judges' evaluations of dynamic gymnastic actions (Exp. 2)

In Experiment 2, our prime interest was to test whether directional bias occurs within the groups of laypeople and judges, respectively. To this end, for each evaluation criterion we compared mean ratings for actions oriented left-to-right vs. right-to-left separately within laypeople and judges.

Here, we additionally ran two-factorial mixed ANOVAs on ratings as a function of the factors action orientation (left-to-right vs. right-to-left) and group (laypeople vs. judges). The descriptive statistics and results from inferential tests are summarized in Table S3 below. As becomes clear from the data, the most relevant differences occurred between laypeople and judges (i.e., main effect for group). Specifically, judges evaluated actions more severe than laypeople as is reflected in lower ratings awarded by judges than laypeople.

Table S3. Laypeople's and judges' evaluations of dynamic gymnastic elements in Exp. 2.

	laypeople		judges		2 (action orientation; AO) x 2 (group) mixed ANOVA			
criterion	*L-to-R*	*R-to-L*	*L-to-R*	*R-to-L*	*effect*	*F*	*p*	η_p^2
technique	7.156 (0.975)	7.031 (1.059)	6.140 (1.113)	6.085 (1.045)	AO	4.155	.044	.042
					group	21.926	< .001	.189
					AO x group	0.649	.422	.007
posture	7.073 (1.035)	6.904 (1.078)	6.183 (1.134)	6.252 (1.158)	AO	1.319	.254	.014
					group	12.194	.001	.115
					AO x group	7.439	.008	.073
aesthetics	6.848 (1.171)	6.765 (1.095)	6.163 (1.060)	6.169 (1.157)	AO	0.635	.427	.007
					group	8.194	.005	.080
					AO x group	0.858	.357	.009
overall	7.138 (1.025)	7.015 (1.048)	6.267 (1.063)	6.252 (1.110)	AO	2.263	.136	.024
					group	14.856	< .001	.136
					AO x group	1.405	.239	.015

Note: *df* = (1, 94) for all analyses.

Performance evaluation in all trials (dynamic & stationary gymnastic elements; Exp. 2)

In Experiment 2, we presented videos of both dynamic and stationary gymnastic elements. As outlined in the main text, our focus was on the analysis of directionality in the evaluation of dynamic gymnastic elements as we expected that directionality would especially occur for these actions (Friedrich et al., 2014; Maass et al., 2007) rather than stationary gymnastic elements where a gymnast does not alter her position. Stationary gymnastic elements were included in the experiment as "fillers" (to familiarize with task and footage) before a block of dynamic gymnastic elements and to enhance variation in stimulus material.

Analyses of mean performance evaluation for actions oriented left-to-right vs. right-to-left run separately for each criterion did not reveal evidence of a reliable and systematic directionality neither in judges nor laypeople. Compared to the results on the evaluations of dynamic gymnastic elements only (as reported in the main text) results did only marginally change in judges, whereas in laypeople the small effects reported in the main text have become even smaller, thus not providing any indication of directionality (see Table S4).

Table S4. Results from one-tailed paired t-tests on evaluations in all trials in Exp. 2.

		M (SD)		Classical[a]				Bayesian[b]	
group	criterion	*L-to-R*	*R-to-L*	*t*	*p*	d_{unb}	*95% CI*	BF_{+0}	error %
judges	technique	6.254 (1.093)	6.177 (1.064)	1.678	.050	0.070	(-0.014, 0.155)	1.088	~4.665e[-8]
	posture	6.273 (1.146)	6.310 (1.172)	-0.960	.928	-0.031	(-0.096, 0.033)	0.086	~6.697e[-8]
	aesthetics	6.194 (1.125)	6.212 (1.150)	-0.357	.639	-0.016	(-0.104, 0.072)	0.121	~7.171e[-8]
	overall	6.335 (1.079)	6.328 (1.101)	0.159	.437	0.006	(-0.068, 0.080)	0.178	~7.198e[-8]
laypeople	technique	7.064 (0.949)	6.986 (0.957)	1.259	.107	0.081	(-0.047, 0.210)	0.583	~6.059e[-8]
	posture	7.012 (0.985)	6.917 (0.950)	1.379	.087	0.097	(-0.043, 0.239)	0.691	~5.711e[-8]
	aesthetics	6.823 (1.087)	6.738 (1.001)	1.056	.148	0.080	(-0.071, 0.232)	0.447	~6.528e[-8]
	overall	7.077 (0.982)	7.007 (0.947)	1.035	.153	0.072	(-0.066, 0.211)	0.436	~6.569e[-8]

Note: For all tests, the to-be-tested hypothesis was that actions evolving from left-to-right are evaluated better than actions from right-to-left. Accordingly, *p*-values are one-tailed.

[a] For classical t-tests $df = 47$. Effect size d_{unb} and associated 95% confidence intervals were calculated with the Estimation Software for Confidence Intervals (Cumming, 2012).

[b] Bayesian t-tests were conducted using the statistical software JASP (version 0.7.5.6; JASP Team, 2016). A default Cauchy prior width of 0.707 was used.

Association between directionality in individuals' aesthetic preference (Exp. 1) and performance evaluation (Exp. 2)

We also checked for possible linear relationships between individuals' directional bias in aesthetic preferences as revealed in Experiment 1 and rating differences observed in Experiment 2 (dynamic gymnastic elements only) separately for the four performance criteria. Again, albeit respective correlations were consistently positive in laypeople (technique: r = .175, p = .235, BF_{10} = 0.357; posture: r = .216, p = .139, BF_{10} = 0.520; aesthetics: r = .191, p = .193, BF_{10} = 0.409; overall: r = .251, p = .085, BF_{10} = 0.758), but not in judges (technique: r = .011, p = .939, BF_{10} = 0.180; posture: r = -0.43, p = .770, BF_{10} = 0.188; aesthetics: r = -.044, p = .766, BF_{10} = 0.188; overall: r = -.105, p = .479, BF_{10} = 0.230), there was no reliable indication or strength of evidence for an association between the various measures (beta* prior width = 1 for calculations of BF_{10}).

Bayes factors robustness checks

A concern in Bayes factor calculation relates to the subjectivity in setting the value for Cauchy prior width. By default, that value is set to 0.707 in the statistical software *JASP* for Bayesian *t*-statistics (JASP Team, 2016). We used that default value for the calculation of Bayes factor reported in the main manuscript.

To explore the robustness of Bayes factors across a range of Cauchy prior widths, we additionally ran Bayes factor robustness checks in *JASP* separately for laypeople and judges for each action element condition (i.e., gymnastic/non-gymnastic x dynamic/stationary) and overall.

The results from Bayes factor robustness checks are illustrated in Figures S1-6 on the following pages.

Experiment 1. For laypeople, Bayes factors were particularly robust (in terms of classification to evidence categories) in favour of a left-to-right image selection bias for dynamic and stationary non-gymnastic pictures (Fig. S1) as well as for all trials (i.e., overall; Fig. S3A), but not for dynamic gymnastic pictures (Fig. S1). For judges, Bayes factors appear robust in that they consistently indicate evidence in favour of the hypothesis of no left-to-right image selection bias (Fig. S2 & S3B). Also, evidence in favour of the hypothesis of no left-to-right bias in image selection increases with increasing prior width. For the comparison of laypeople and judges, robustness check indicates that Bayes factors do not suggest strong evidence in favour of group differences across different Cauchy prior width (Fig. S4).

Experiment 2. For laypeople, Bayes factors were less robust particularly for the criteria technique and overall (Fig. S5), while for the criterion posture robustness may be accepted. For judges, Bayes factors were generally more robust across criteria (Fig. S6). However, Bayes factors consistently indicate evidence against the hypothesis of better evaluation of "left-to-right" than "right-to-left" dynamic gymnastic elements.

Experiment 1.

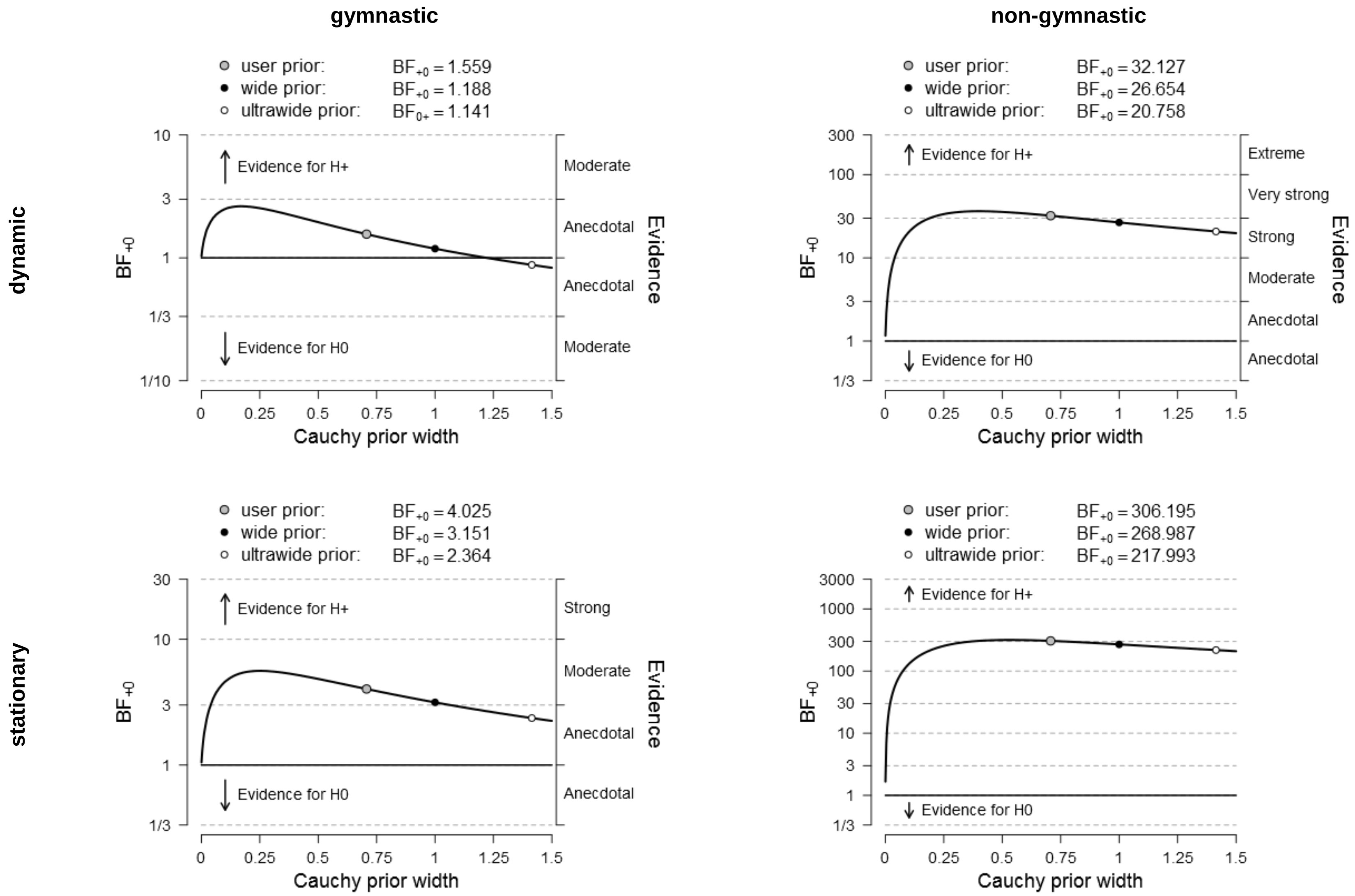

Figure S1. Bayes factor robustness checks in laypeople regarding the directed hypothesis of above random “left-to-right”-image selection separately for the four action element conditions realized in Experiment 1.

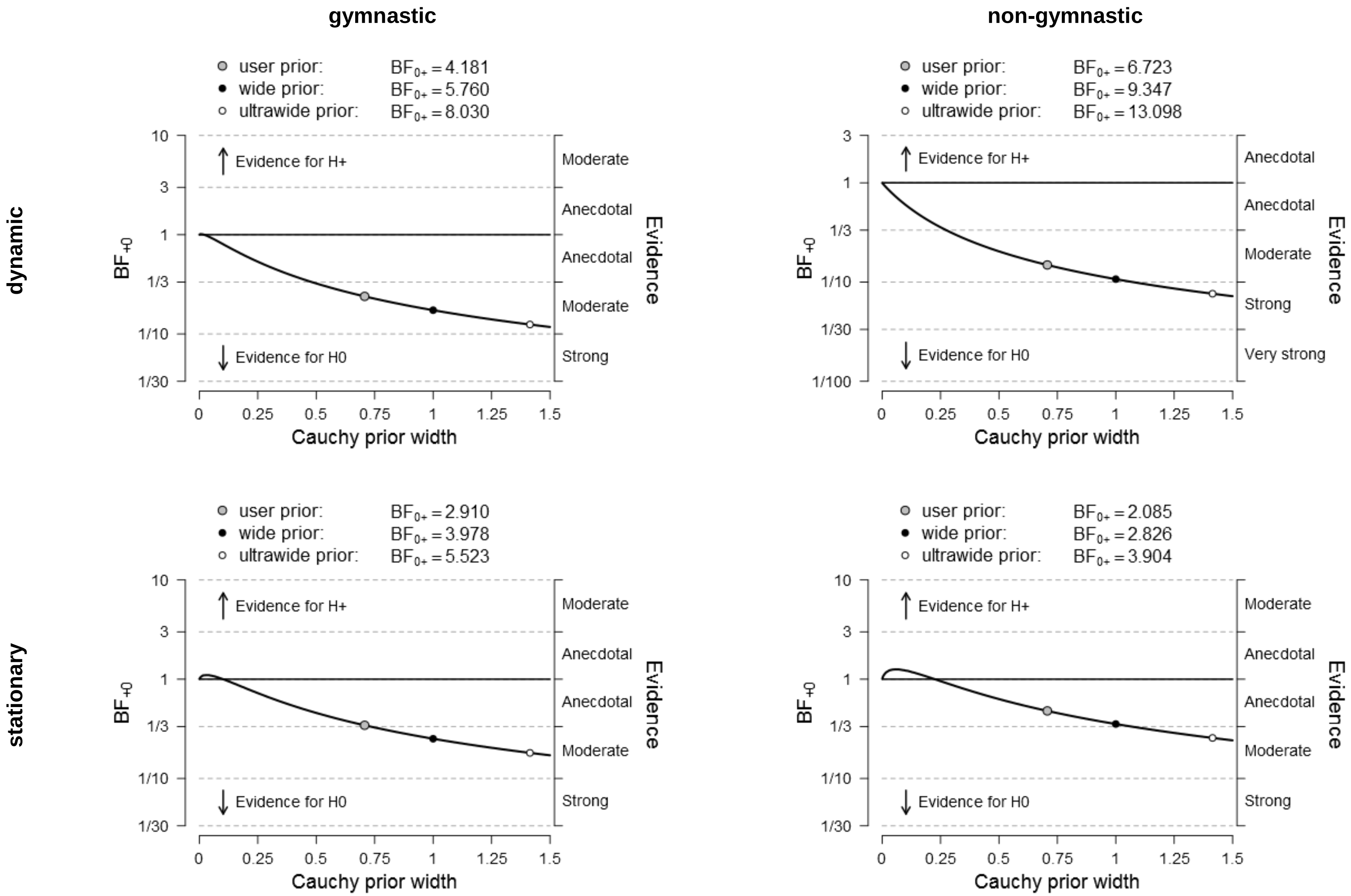

Figure S2. Bayes factor robustness checks in judges regarding the directed hypothesis of above random “left-to-right”-image selection separately for the four action element conditions realized in Experiment 1.

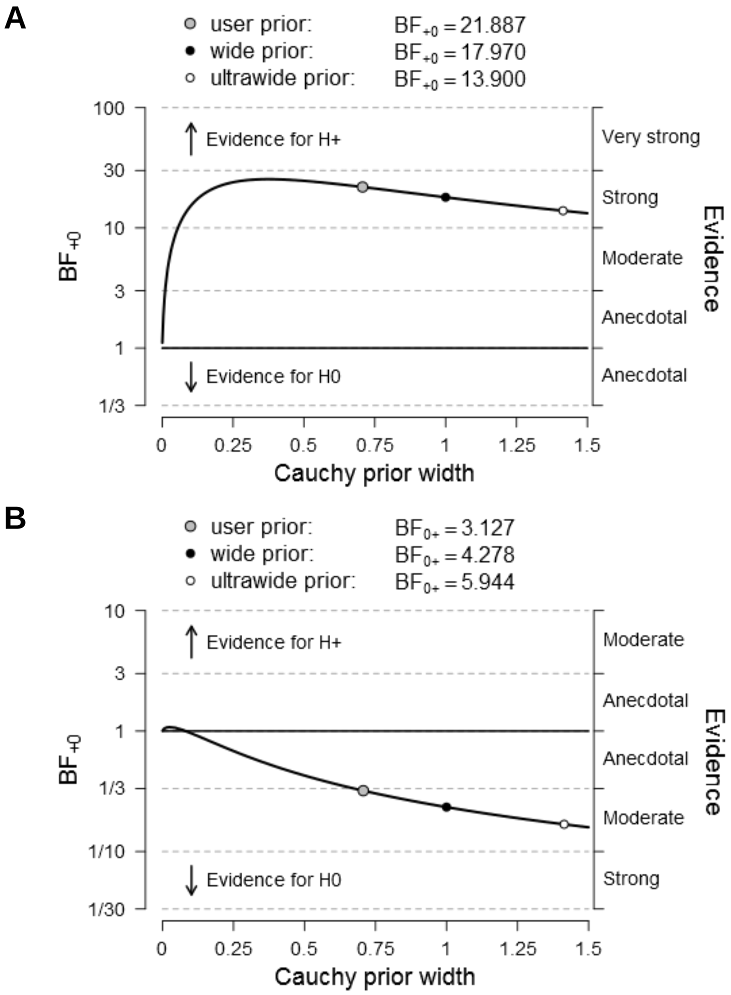

Figure S3. Bayes factor robustness checks in (**A**) laypeople and (**B**) judges regarding the directed hypothesis of above random “left-to-right”-image selection across all trials (“overall”) in Experiment 1.

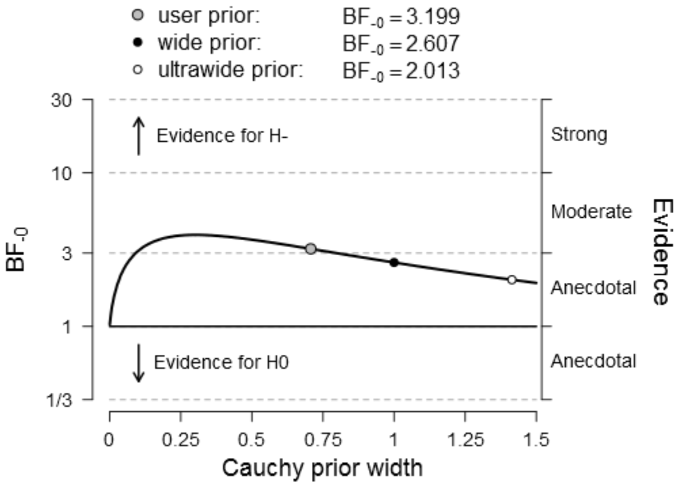

Figure S4. Bayes factor robustness check on the comparison of laypeople vs. judges regarding the directed hypothesis of a group difference in “left-to-right”-image selection across all trials (“overall”) in Experiment 1.

Experiment 2.

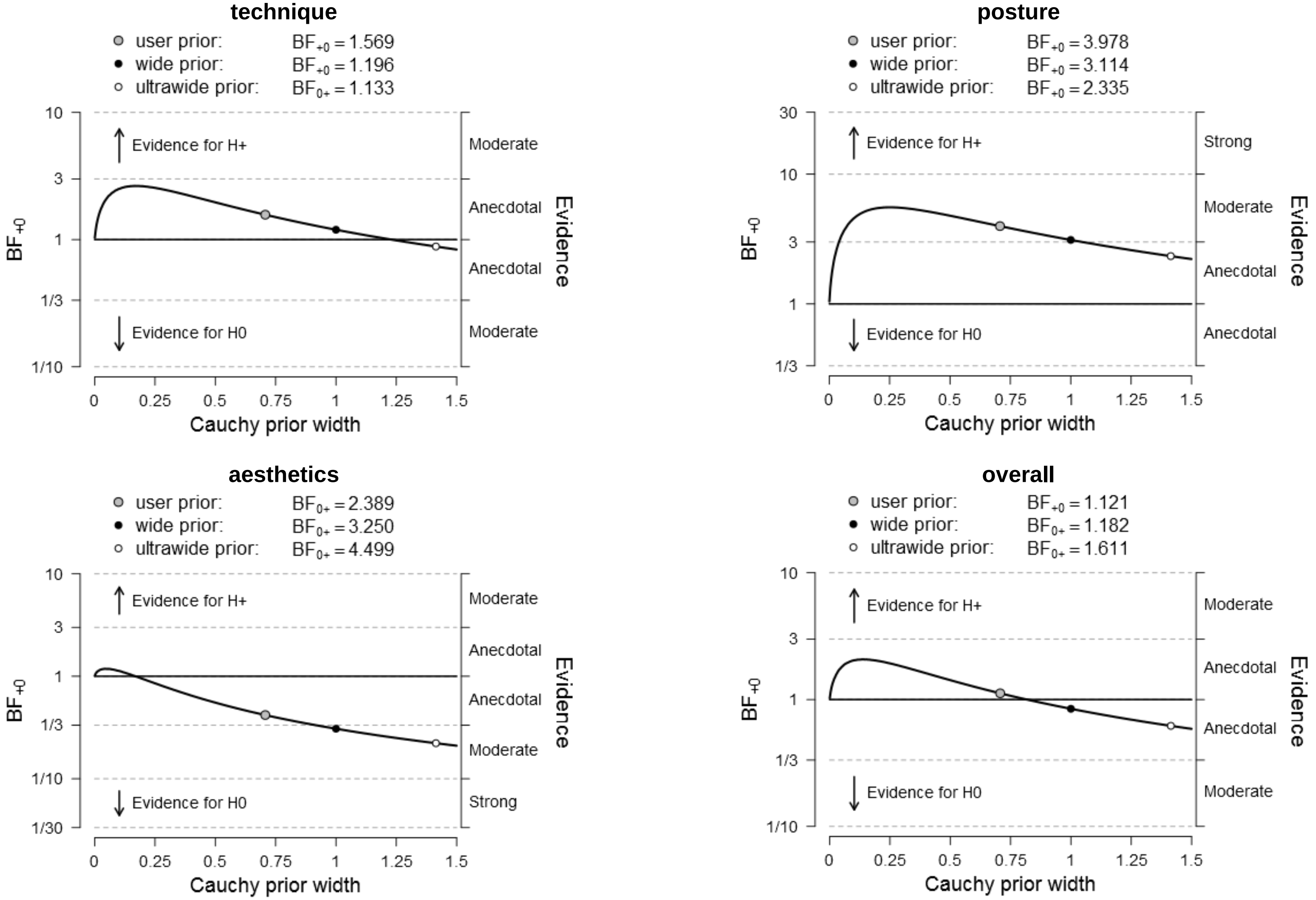

Figure S5. Bayes factor robustness checks in laypeople regarding the directed hypothesis of better evaluation of “left-to-right” than “right-to-left” dynamic gymnastic elements separately for the four evaluation criteria in Experiment 2.

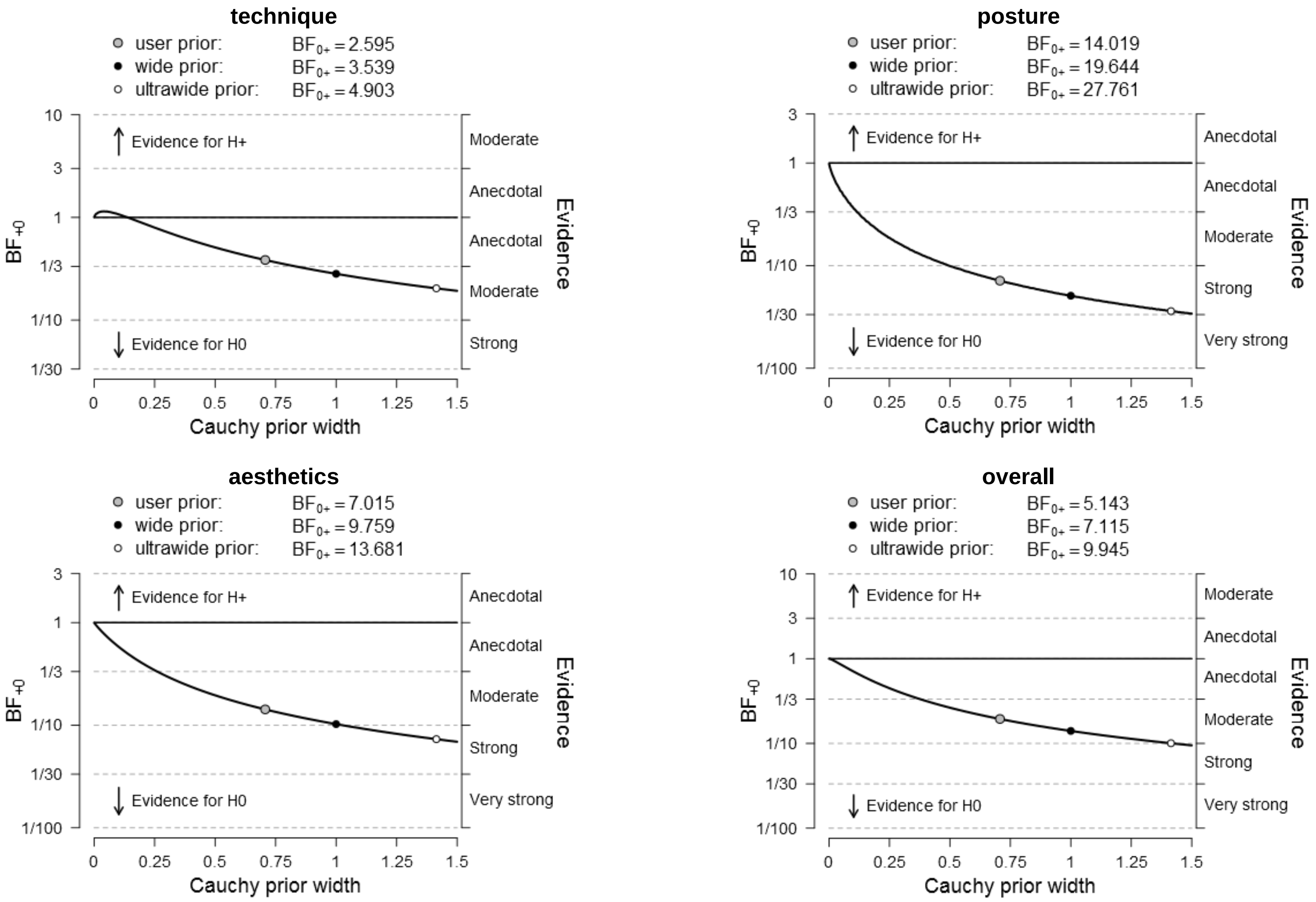

Figure S6. Bayes factor robustness checks in judges regarding the directed hypothesis of better evaluation of "left-to-right" than "right-to-left" dynamic gymnastic elements separately for the four evaluation criteria in Experiment 2.

Sequential analysis of Bayes factors (with robustness check)

In addition to the above Bayes factor robustness checks, we also conducted sequential analyses of Bayes factors (with inclusion of different Cauchy prior width) to illustrate the evidential flow from the first (i.e., n = 1) up to the last participant (n = 48) testing within the groups of laypeople and judges, respectively (see Fig. S7-12 on the following pages). Please note that we did not consider the development of Bayes factors in the conduct of the experiments (e.g., Wagenmakers, Morey, & Lee, 2016) but calculated sequential analyses after termination of data collection.

Experiment 1. For laypeople, sequential analyses indicate quite consistent and increasingly stable evidence in favour of the hypothesis of left-to-right image selection bias for dynamic/stationary non-gymnastic pictures (Fig. S7) and overall (Fig. S9A), but not for dynamic/stationary gymnastic pictures (Fig. S7). For judges, the development of Bayes factors against the hypothesis of a left-to-right image selection bias was relatively stable, though predominantly just in the low evidence categories anecdotal and moderate (Fig. S8 & S9B). For the comparison of laypeople and judges, sequential analysis indicates variability of evidence in favour or against the hypothesis of a group difference around a Bayes factor of 1 (Fig. S10).

Experiment 2. For laypeople, sequential analysis indicates clear variability in the development of Bayes factors that are consistently in favour of the hypothesis of better evaluation of “left-to-right” than “right-to-left” gymnastic elements for the criterion posture (Fig. S11). For the other criteria, Bayes factors were relatively stable around a value of 1 (technique, overall) or between 1 and 1/3 (aesthetics). For judges, with the exception of technique, sequential analyses indicate stable development of Bayes factors against the hypothesis of better evaluation of “left-to-right” than “right-to-left” gymnastic elements (moderate to strong evidence categories), particularly for posture and aesthetics (Fig. S12).

Experiment 1.

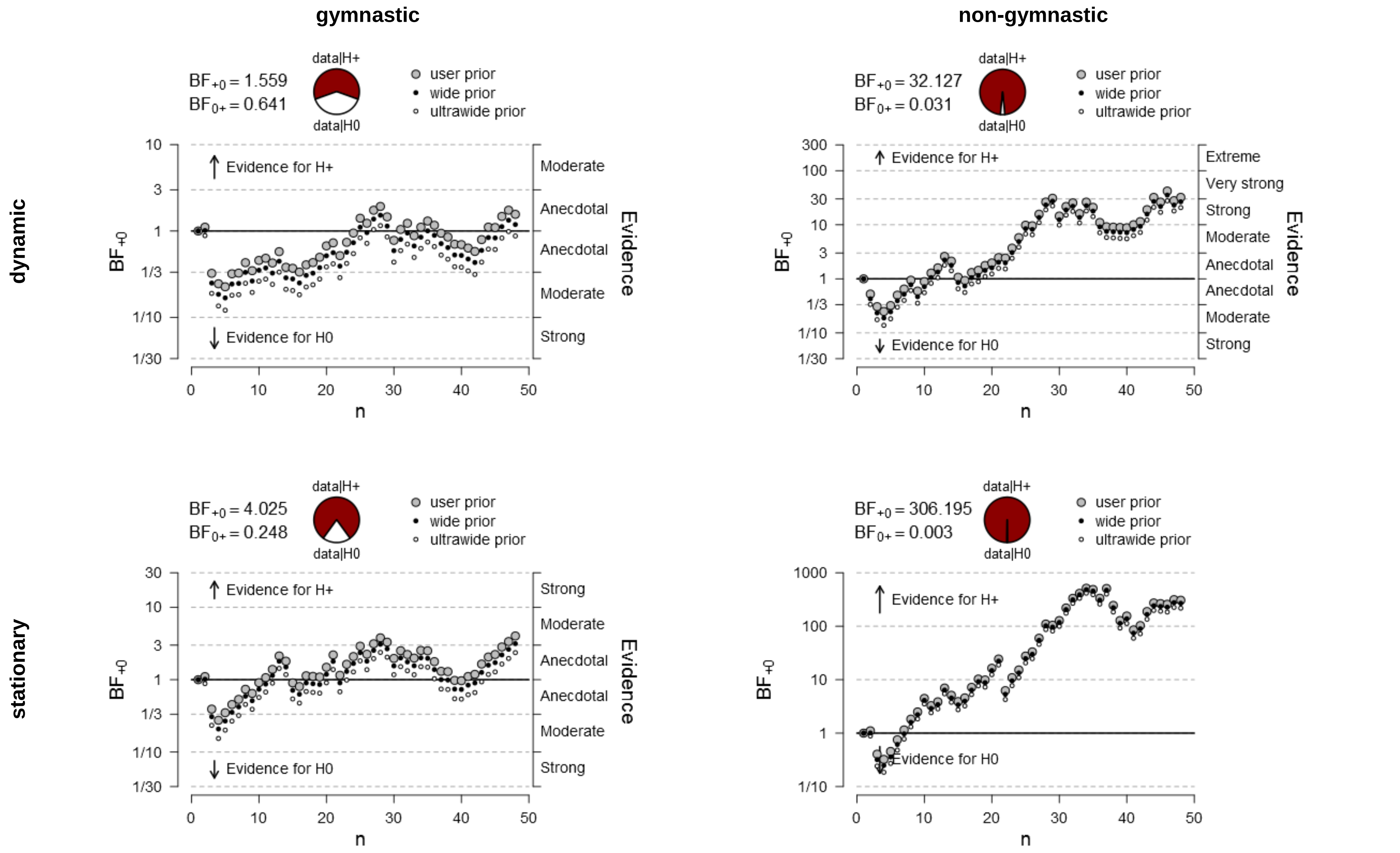

Figure S7. Sequential analysis of Bayes factors (with robustness check) as a function of sample size *n* in laypeople regarding the directed hypothesis of above random "left-to-right"-image selection separately for the four action element conditions realized in Experiment 1.

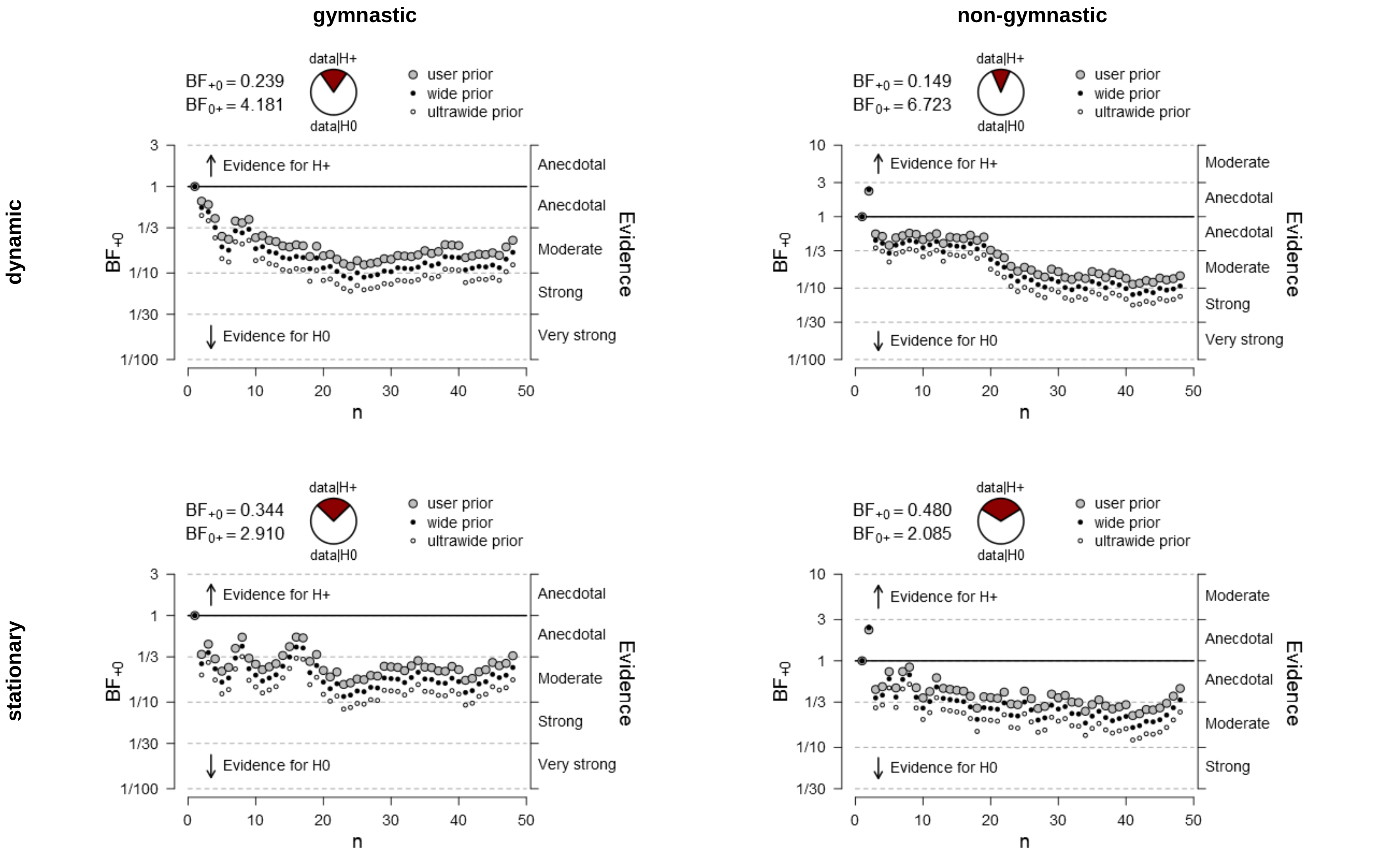

Figure S8. Sequential analysis of Bayes factors (with robustness check) as a function of sample size *n* in judges regarding the directed hypothesis of above random “left-to-right”-image selection separately for the four action element conditions realized in Experiment 1.

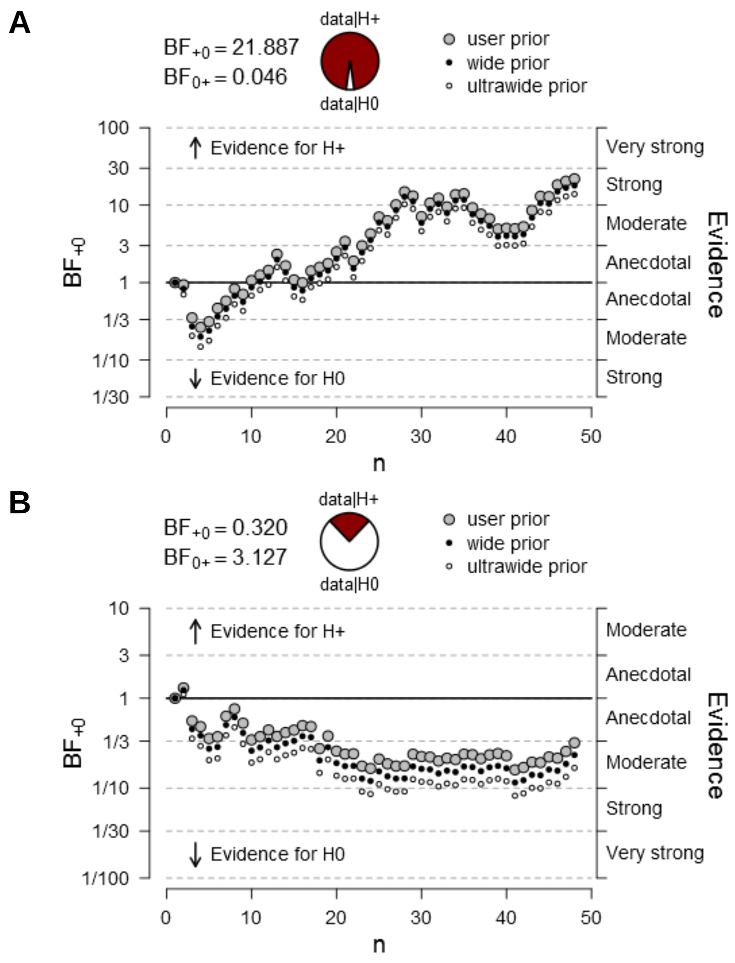

Figure S9. Sequential analysis of Bayes factors (with robustness check) as a function of sample size *n* in (**A**) laypeople and (**B**) judges regarding the directed hypothesis of above random "left-to-right"-image selection across all trials ("overall") in Experiment 1.

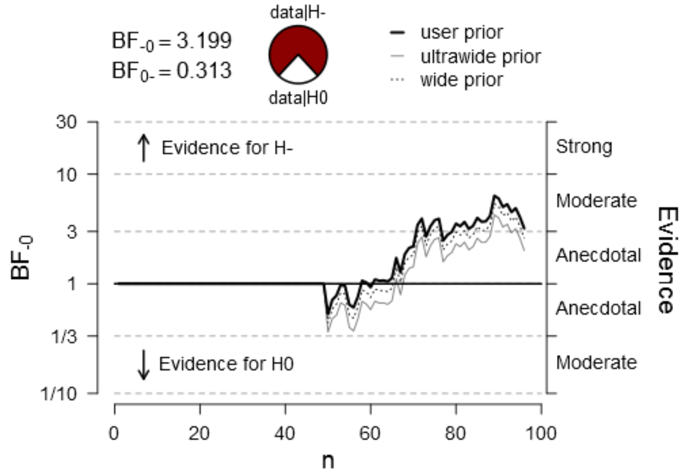

Figure S10. Sequential analysis of Bayes factors (with robustness check) as a function of sample size *n* on the comparison of laypeople vs. judges regarding the directed hypothesis of a group difference in "left-to-right"-image selection across all trials ("overall") in Experiment 1.

Experiment 2.

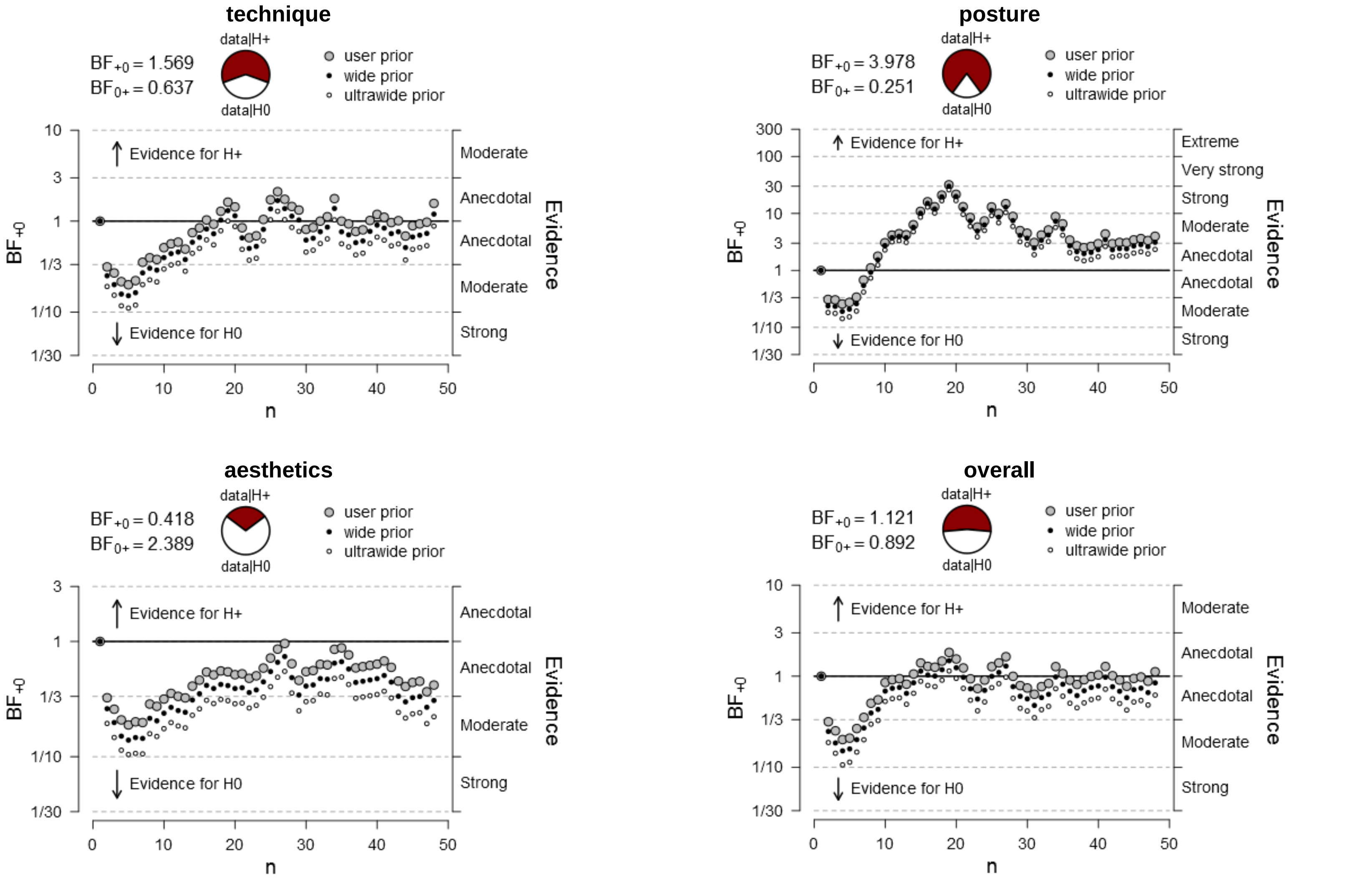

Figure S11. Sequential analysis of Bayes factors (with robustness check) as a function of sample size *n* in laypeople regarding the directed hypothesis of better evaluation of "left-to-right" than "right-to-left" dynamic gymnastic elements separately for the four evaluation criteria in Experiment 2.

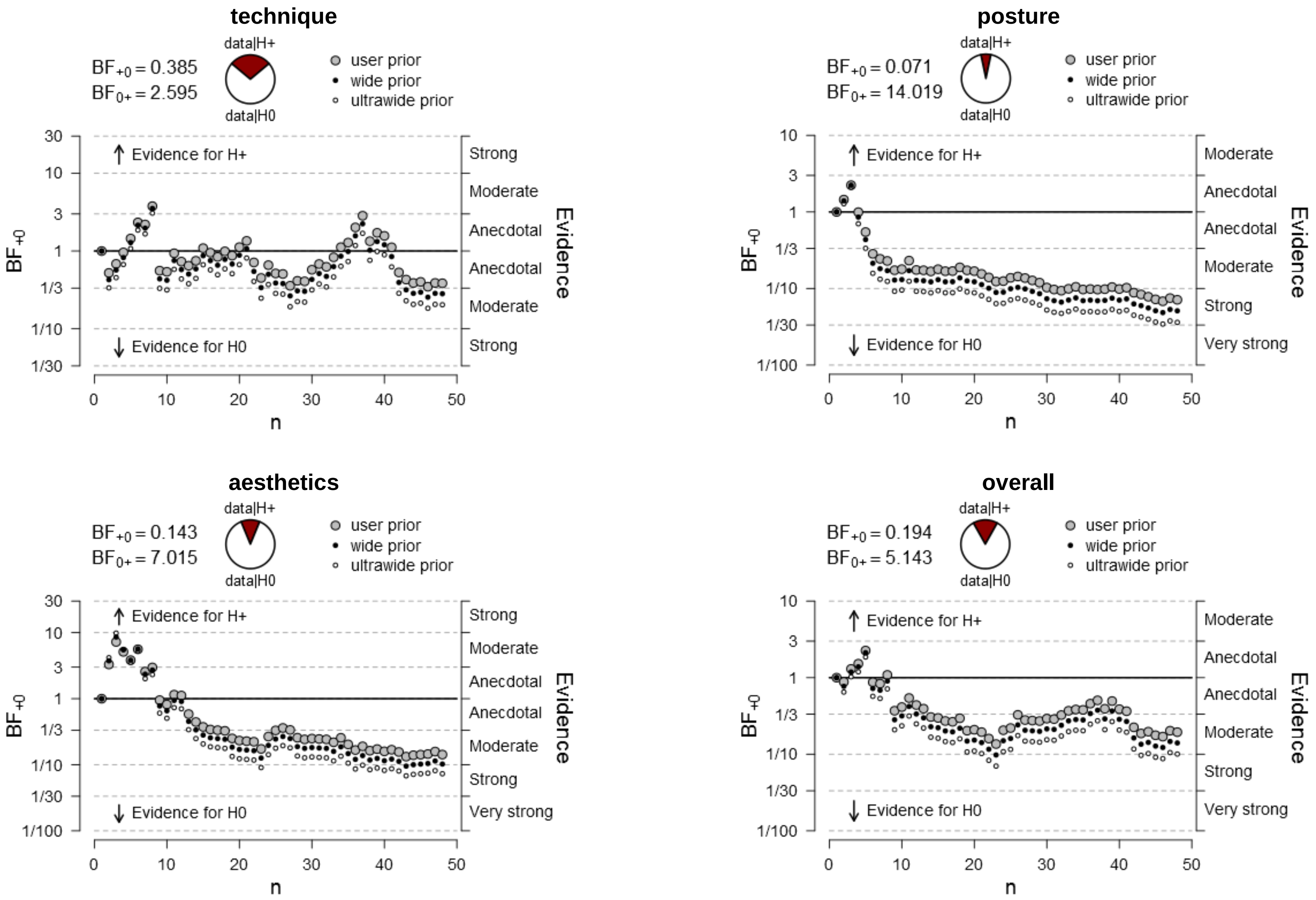

Figure S12. Sequential analysis of Bayes factors (with robustness check) as a function of sample size *n* in judges regarding the directed hypothesis of better evaluation of “left-to-right” than “right-to-left” dynamic gymnastic elements separately for the four evaluation criteria in Experiment 2.

References

Casasanto, D. (2009). Embodiment of abstract concepts: Good and bad in right- and left-handers. *Journal of Experimental Psychology: General, 138*(3), 351-367. doi:10.1037/a0015854

Chokron, S., & De Agostini, M. (2000). Reading habits influence aesthetic preference. *Cognitive Brain Research, 10*(1-2), 45-49. doi:10.1016/s0926-6410(00)00021-5

Cumming, G. (2012). *Understanding the new statistics: Effect sizes, confidence intervals, and meta-analysis*. New York: Routledge.

Faul, F., Erdfelder, E., Lang, A.-G., & Buchner, A. (2007). G*Power 3: A flexible statistical power analysis program for the social, behavioral, and biomedical sciences. *Behavior Research Methods, 39*(2), 175-191. doi:10.3758/BF03193146

Friedrich, T. E., Harms, V. L., & Elias, L. J. (2014). Dynamic stimuli: Accentuating aesthetic preference biases. *Laterality: Asymmetries of Body, Brain and Cognition, 19*(5), 549-559. doi:10.1080/1357650x.2014.886585

Ishii, Y., Okubo, M., Nicholls, M. E. R., & Imai, H. (2011). Lateral biases and reading direction: A dissociation between aesthetic preference and line bisection. *Brain and Cognition, 75*(3), 242-247. doi:10.1016/j.bandc.2010.12.005

JASP Team. (2016). JASP (Version 0.7.5.6). Retrieved from https://jasp-stats.org

Maass, A., Pagani, D., & Berta, E. (2007). How beautiful is the goal and how violent is the fistfight? Spatial bias in the interpretation of human behavior. *Social Cognition, 25*(6), 833-852. doi:10.1521/soco.2007.25.6.833

Wagenmakers, E.-J., Morey, R. D., & Lee, M. D. (2016). Bayesian benefits for the pragmatic researcher. *Current Directions in Psychological Science, 25*(3), 169-176. doi:10.1177/0963721416643289
