# Supplementary material for: Directionality in Aesthetic Judgments and Performance Evaluation: Sport Judges and Laypeople Compared
Source: Front Psychol. 2017 Dec 5;8:2109. doi: 10.3389/fpsyg.2017.02109 (PMC5723409; doi:10.3389/fpsyg.2017.02109)
Supplement: SUPPLEMENTARY MATERIAL S2 — Images presented in Experiment 1. [file Supplementary_Material_S2.PDF]

Experiment 1. Stationary gymnastic elements.

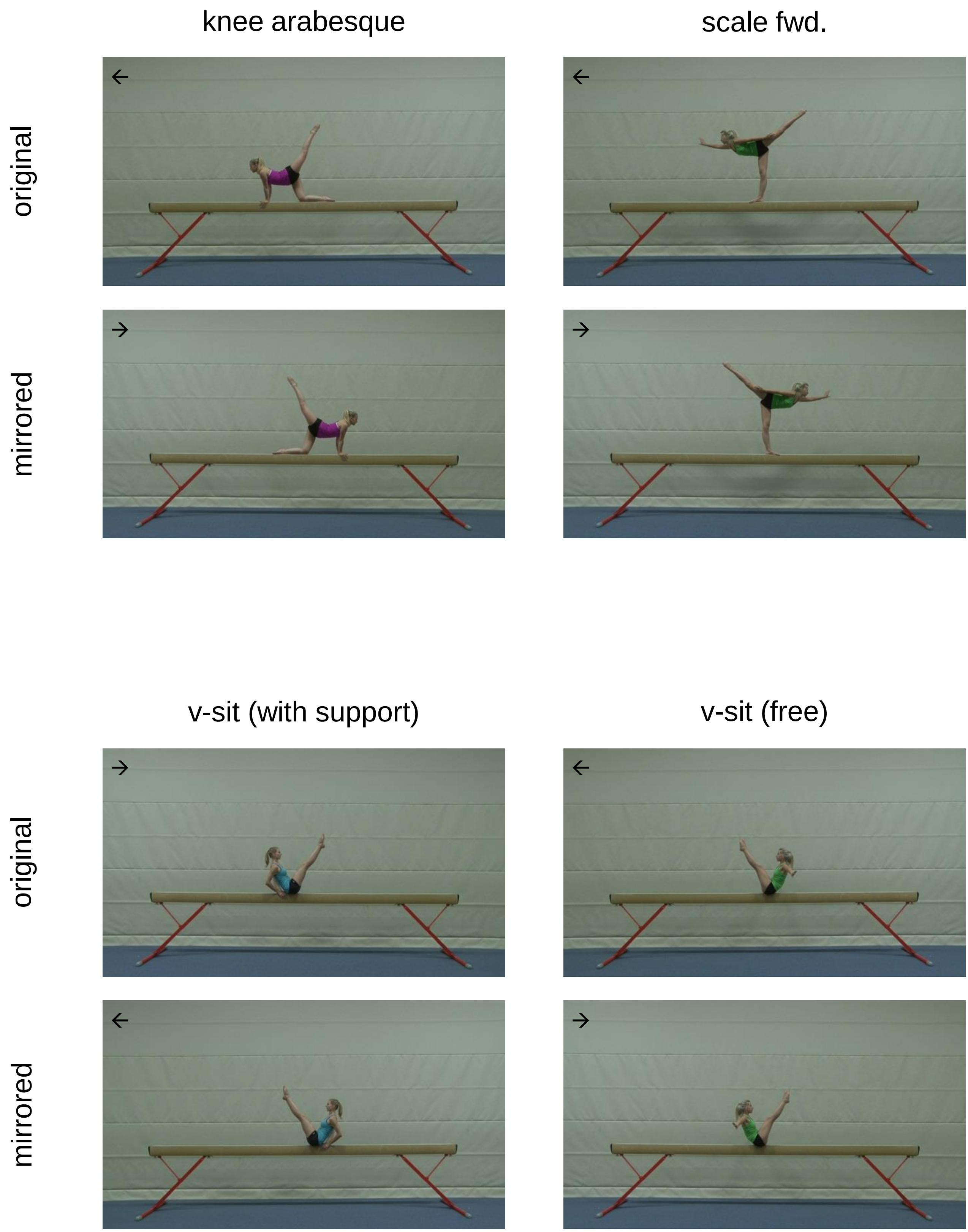

Arrows in the top left corner of pictures indicate the coding of horizontal orientation:
"→" = left-to-right; "←" = right-to-left

Experiment 1. Stationary gymnastic elements.

front lying support

straddle support

original

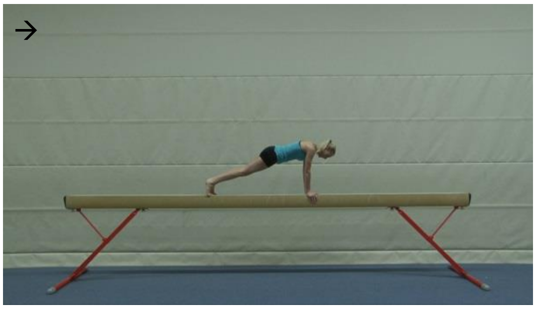

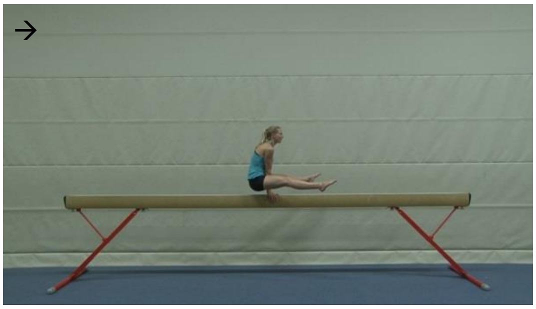

mirrored

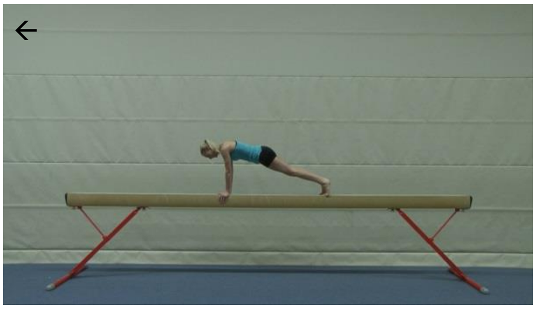

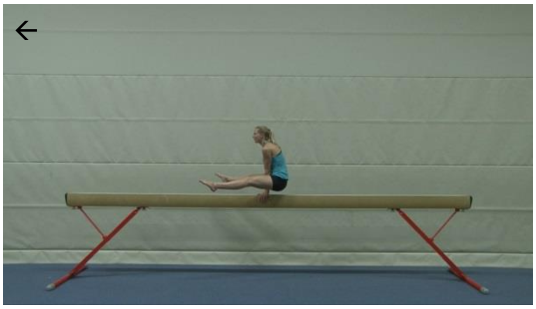

handstand

pose (standing)

original

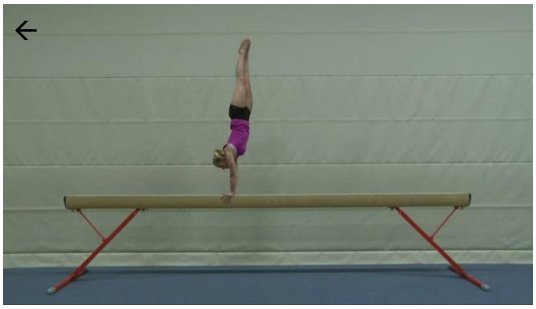

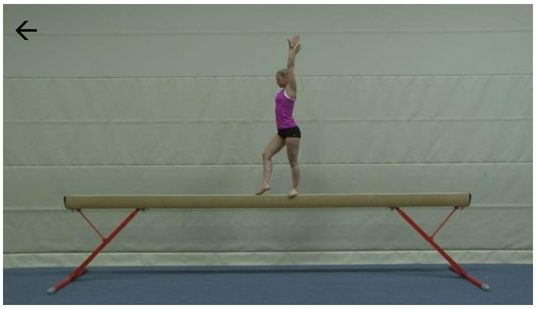

mirrored

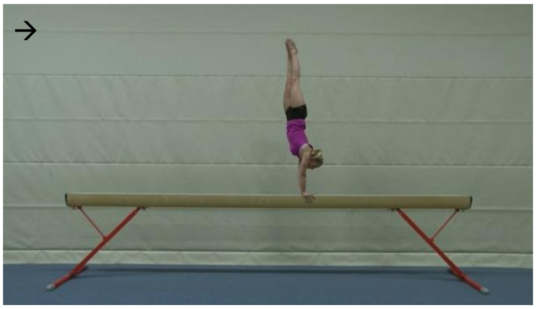

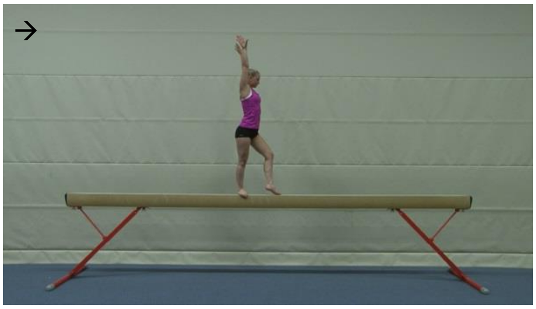

Arrows in the top left corner of pictures indicate the coding of horizontal orientation:
“→” = left-to-right; “←” = right-to-left

Experiment 1. Stationary gymnastic elements.

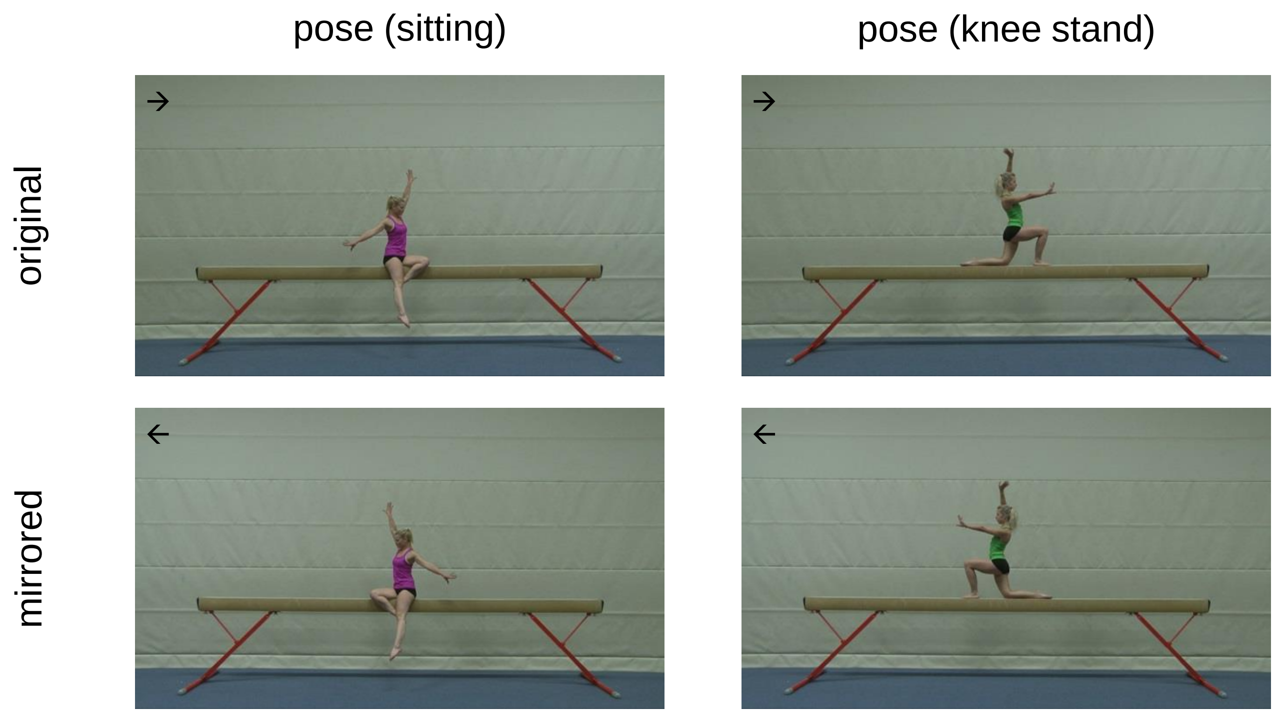

Arrows in the top left corner of pictures indicate the coding of horizontal orientation:
"→" = left-to-right; "←" = right-to-left

Experiment 1. Dynamic gymnastic elements.

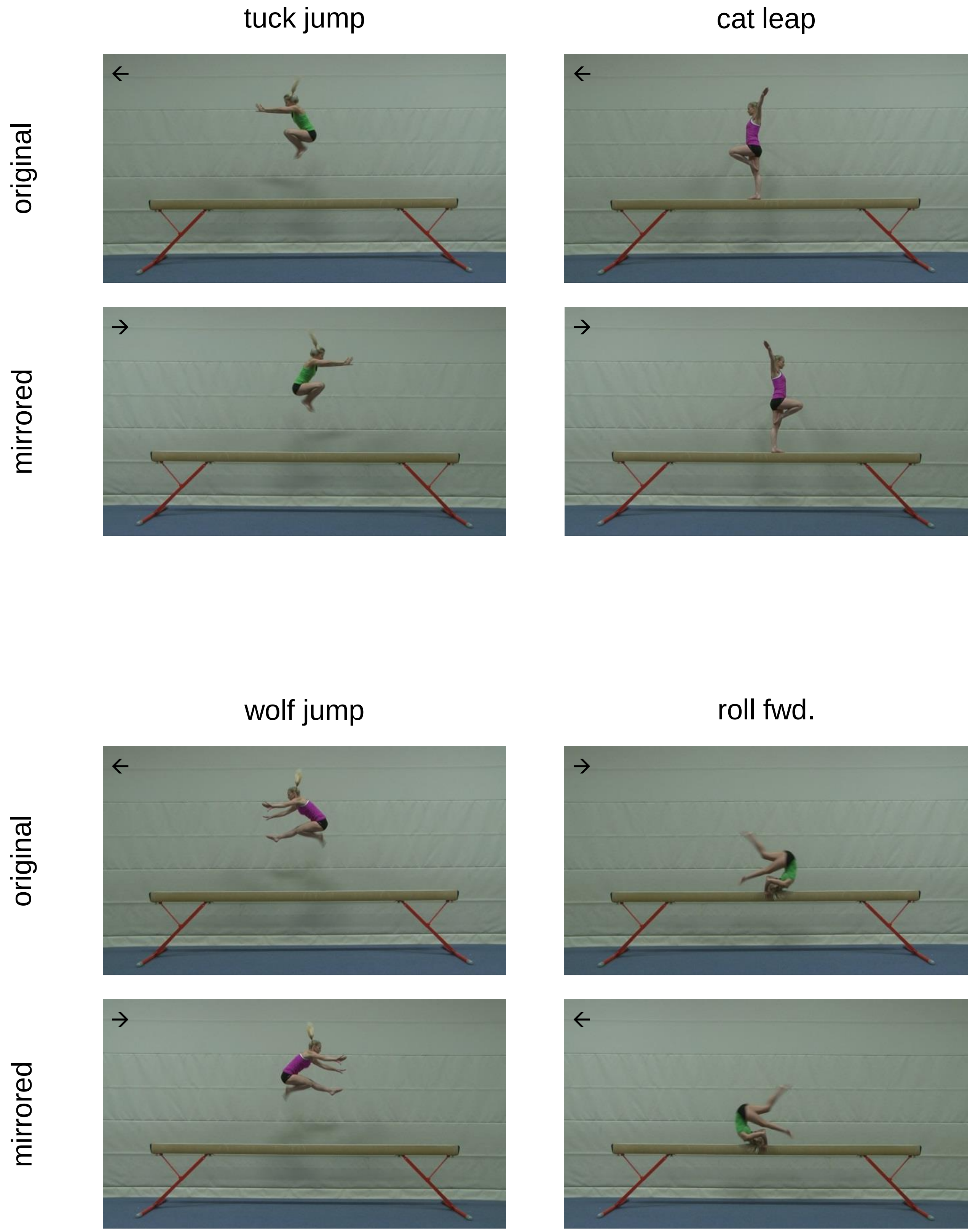

Arrows in the top left corner of pictures indicate the coding of horizontal orientation:
"→" = left-to-right; "←" = right-to-left

Experiment 1. Dynamic gymnastic elements.

split leap fwd. | stretched jump

original

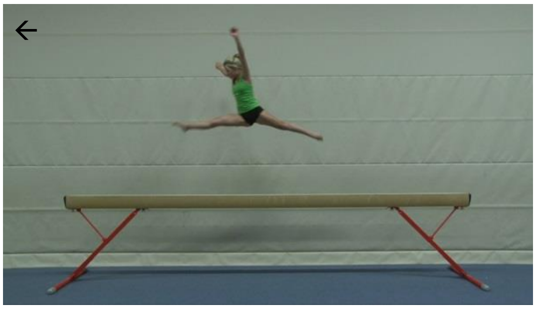

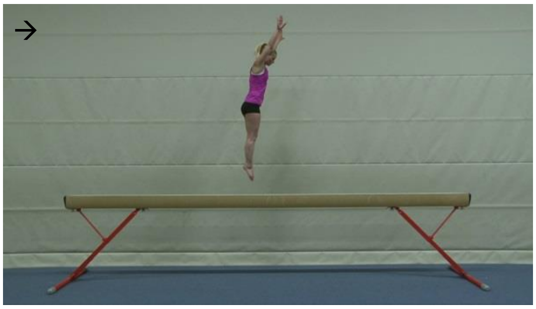

mirrored

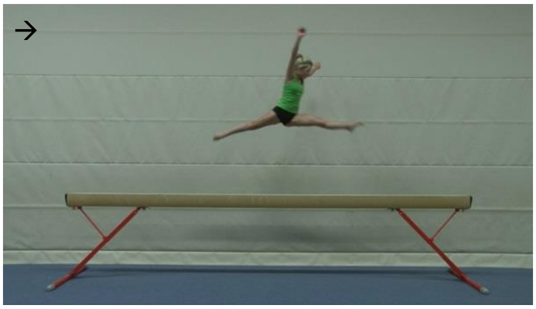

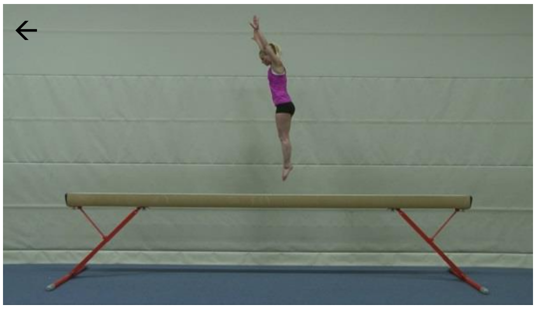

stretched jump with leg change | step-close steps fwd.

original

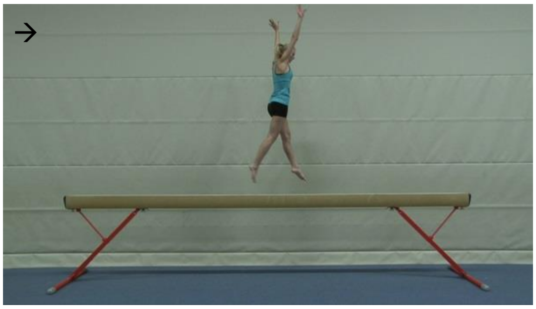

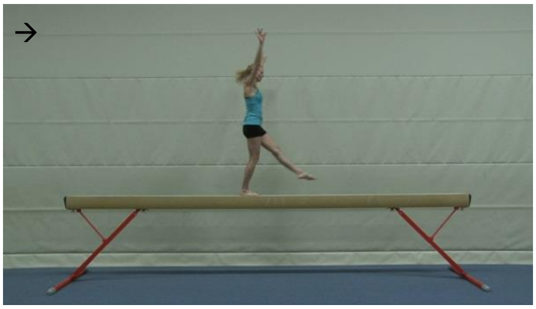

mirrored

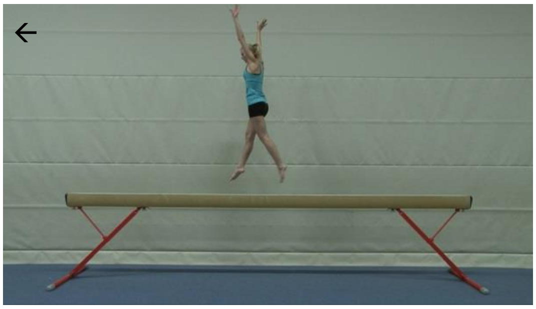

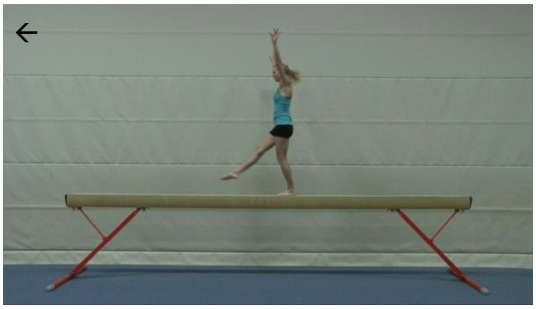

Arrows in the top left corner of pictures indicate the coding of horizontal orientation:
"→" = left-to-right; "←" = right-to-left

Experiment 1. Dynamic gymnastic elements.

turn on one foot (360°) | scissors leap fwd.

original / mirrored

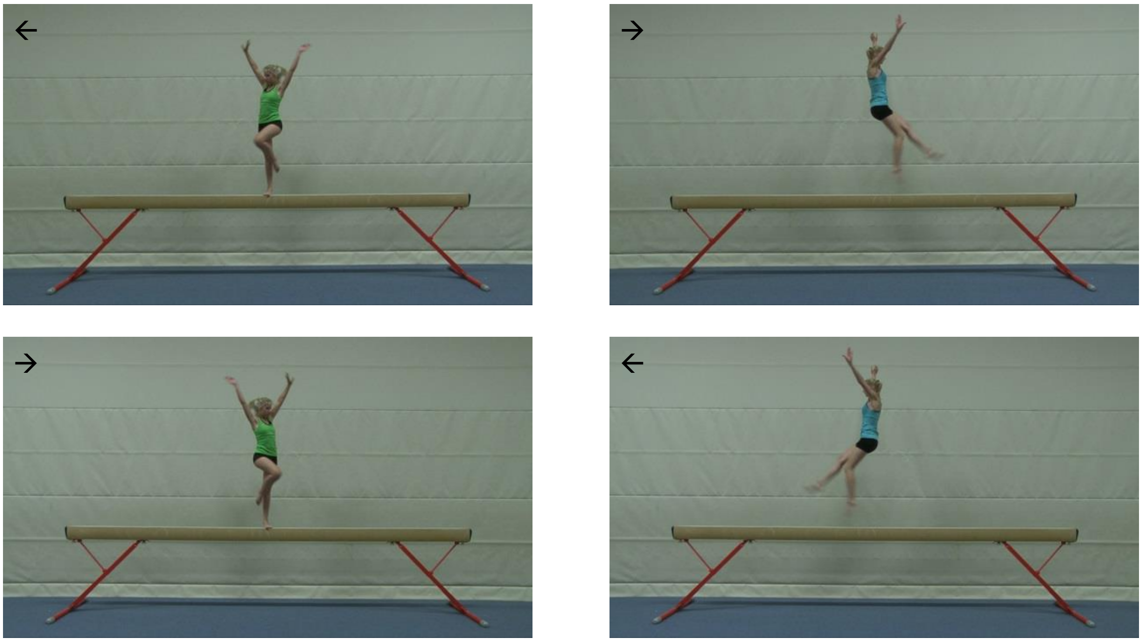

Arrows in the top left corner of pictures indicate the coding of horizontal orientation:
“→” = left-to-right; “←” = right-to-left

Experiment 1. Stationary non-gymnastic elements.

reading a book

looking through field glasses

original

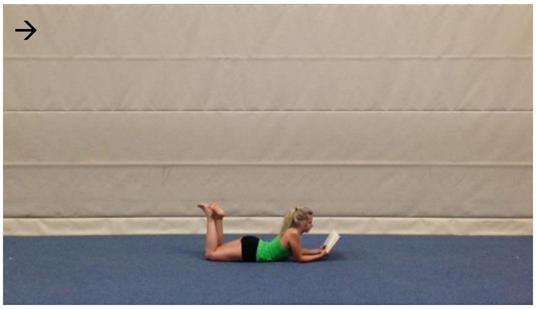

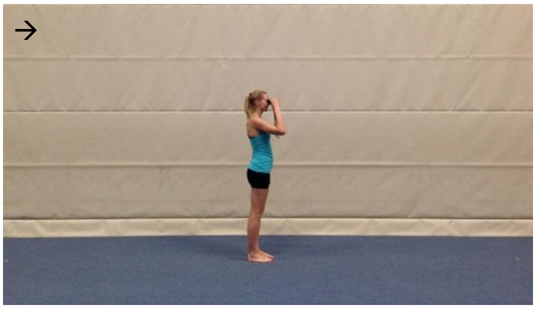

mirrored

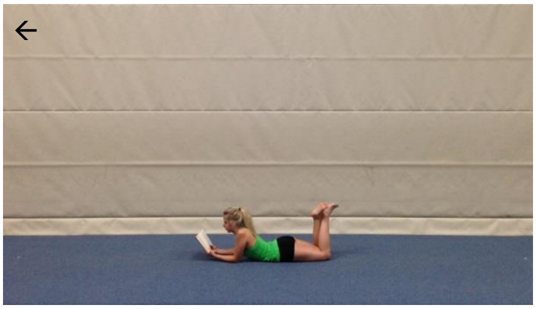

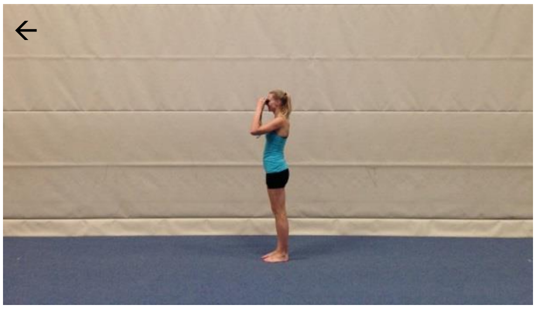

browsing through a photo album

relaxing on a deckchair

original

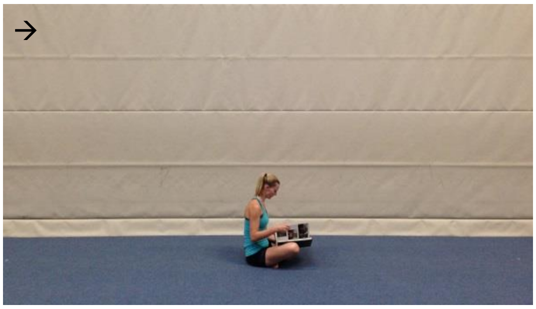

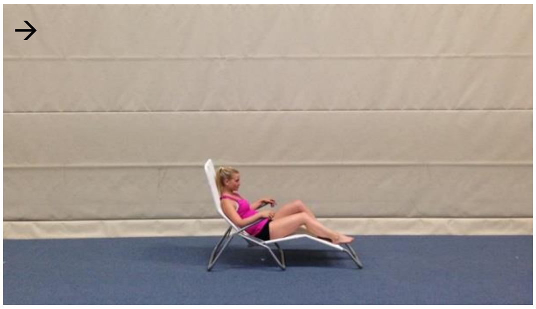

mirrored

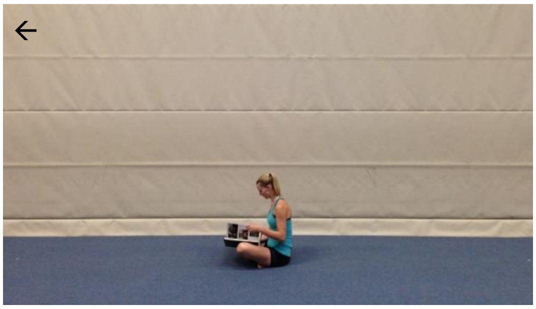

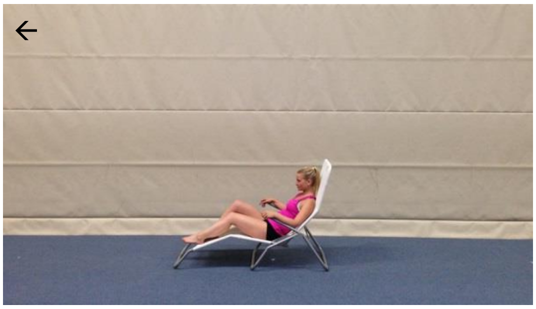

Arrows in the top left corner of pictures indicate the coding of horizontal orientation:
"→" = left-to-right; "←" = right-to-left

Experiment 1. Stationary non-gymnastic elements.

raising a trophy

drinking

original

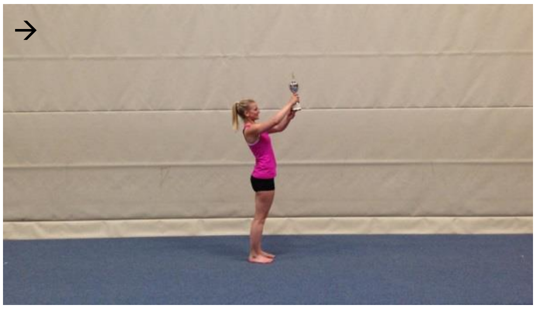

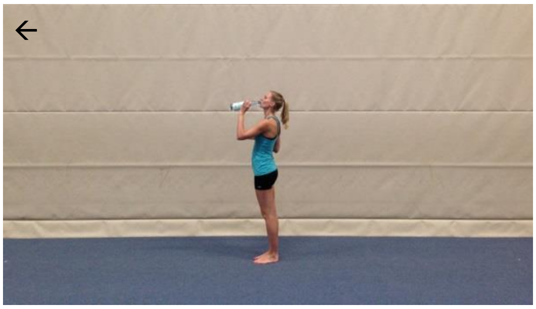

mirrored

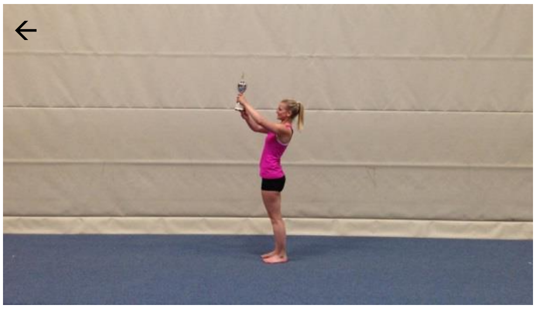

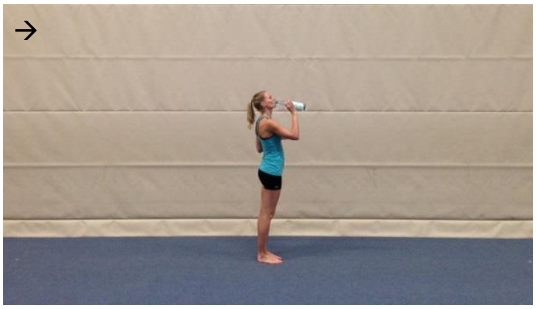

working on a notebook

doing push-ups

original

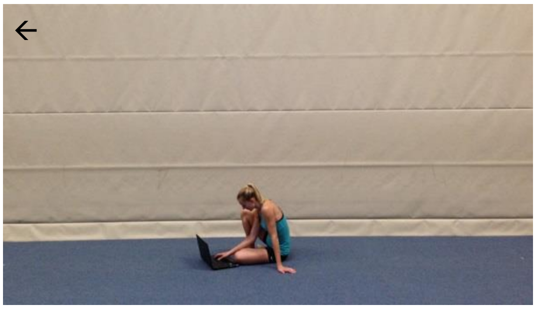

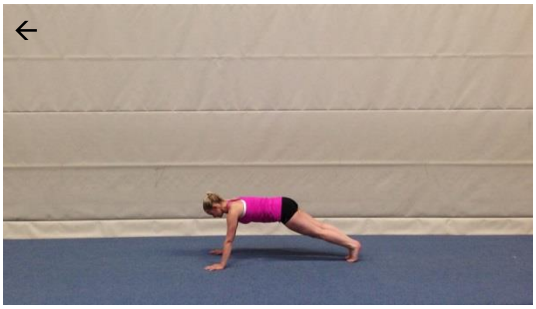

mirrored

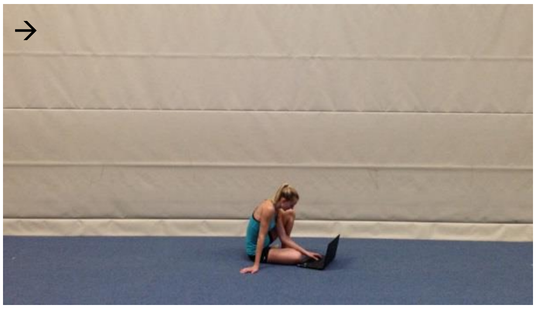

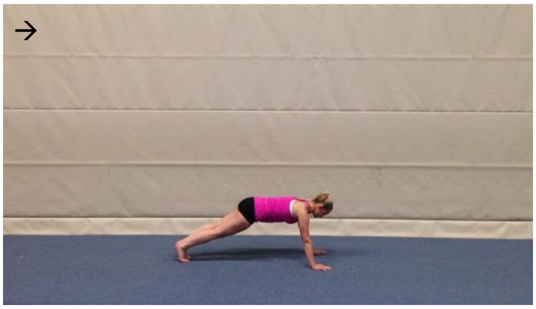

Arrows in the top left corner of pictures indicate the coding of horizontal orientation:
"→" = left-to-right; "←" = right-to-left

Experiment 1. Stationary non-gymnastic elements.

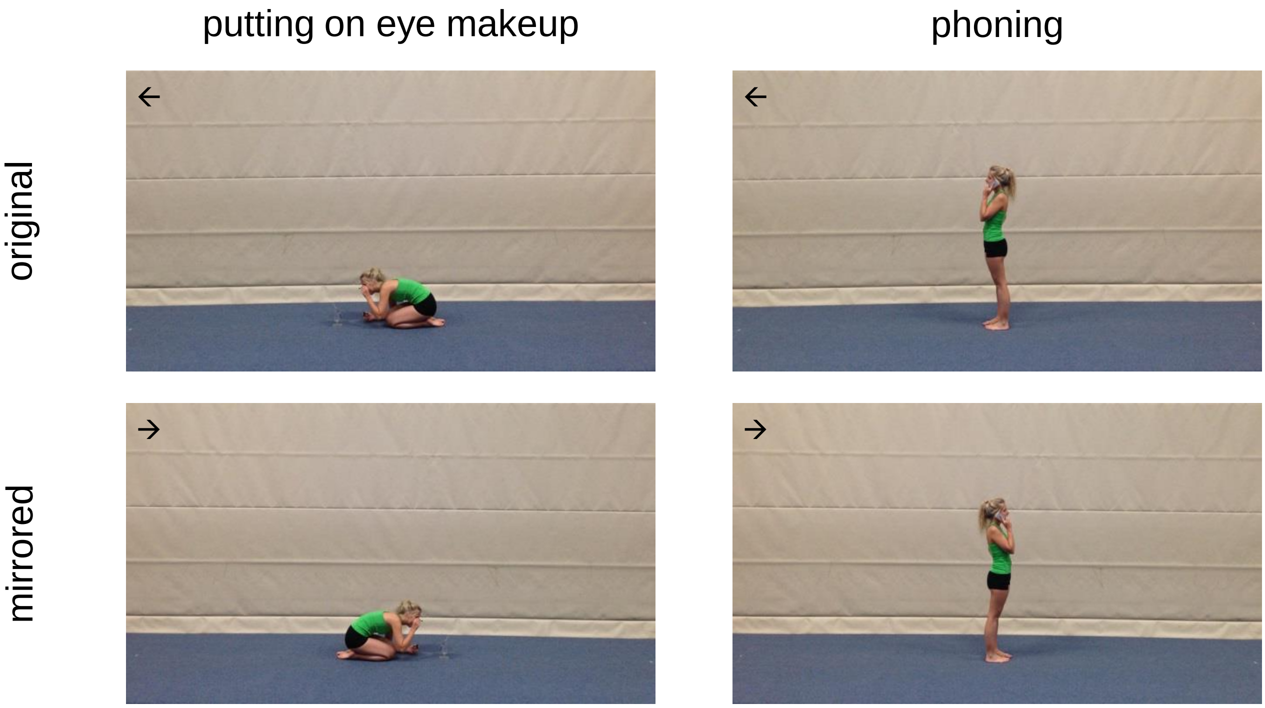

Arrows in the top left corner of pictures indicate the coding of horizontal orientation:
“→” = left-to-right; “←” = right-to-left

Experiment 1. Dynamic non-gymnastic elements.

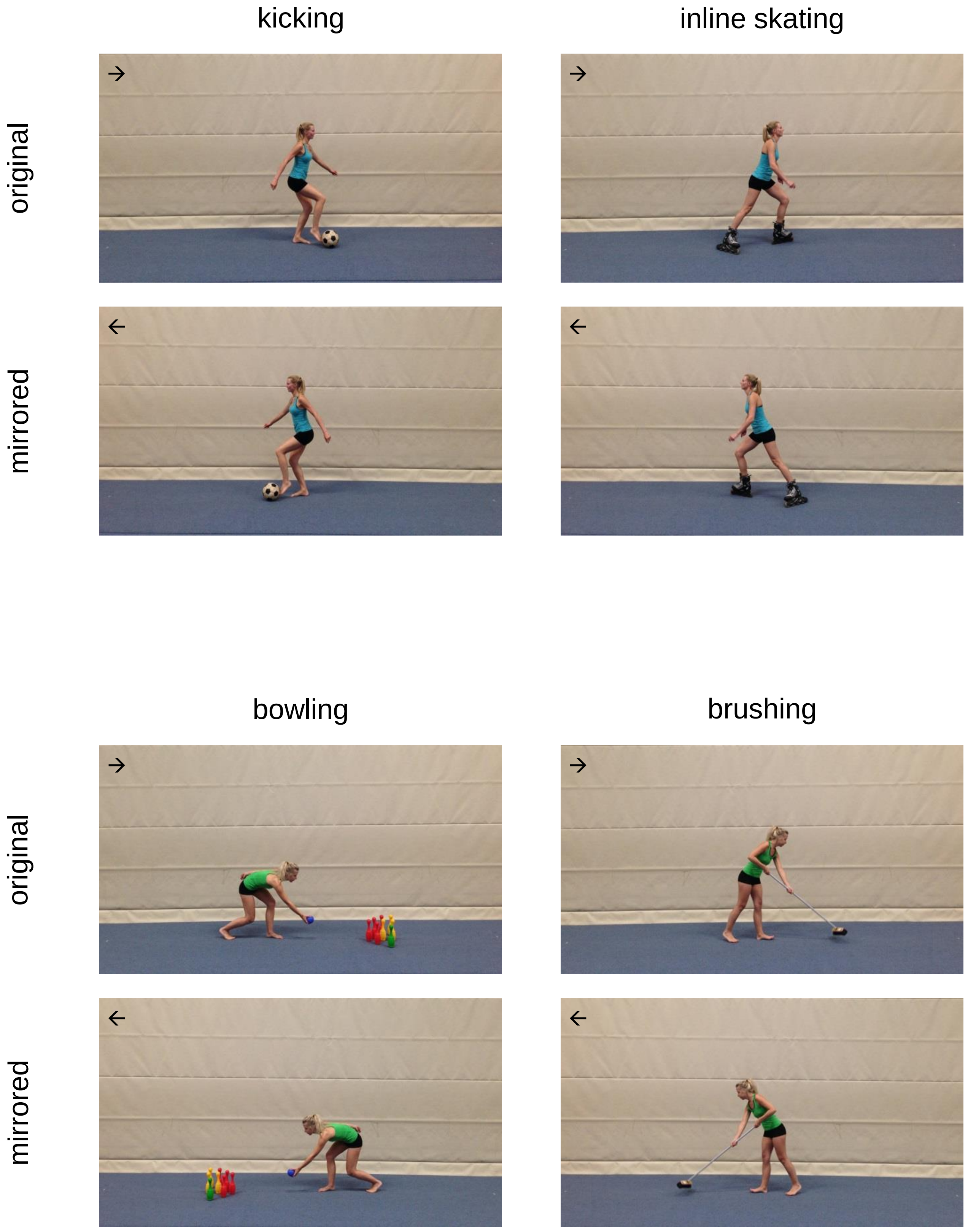

Arrows in the top left corner of pictures indicate the coding of horizontal orientation:
"→" = left-to-right; "←" = right-to-left

Experiment 1. Dynamic non-gymnastic elements.

original / mirrored

riding a scooter

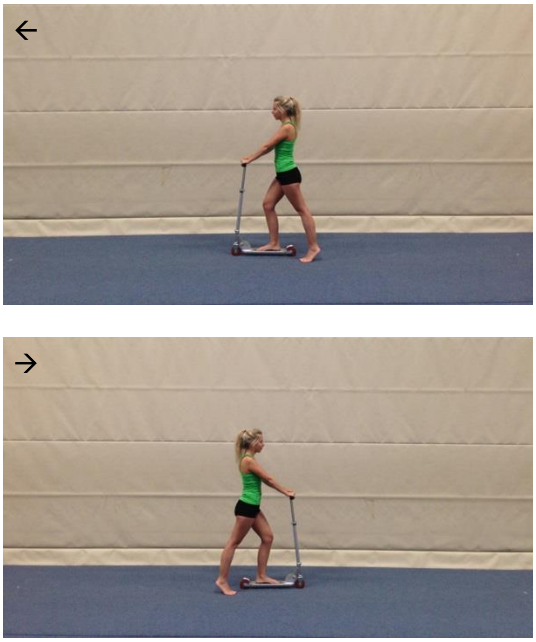

jogging

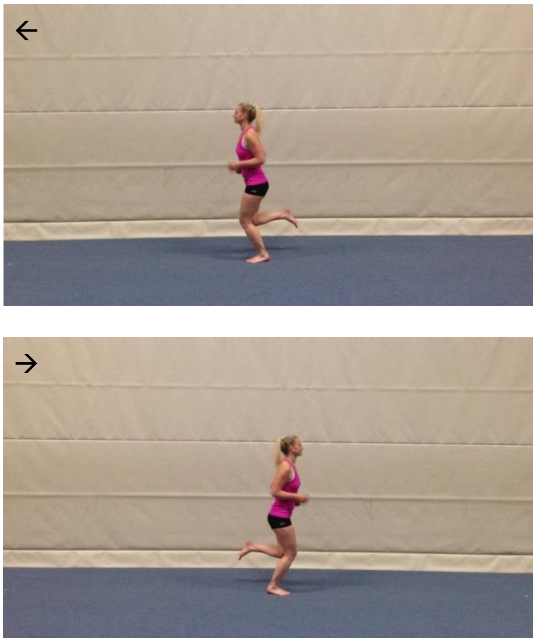

original / mirrored

playing badminton

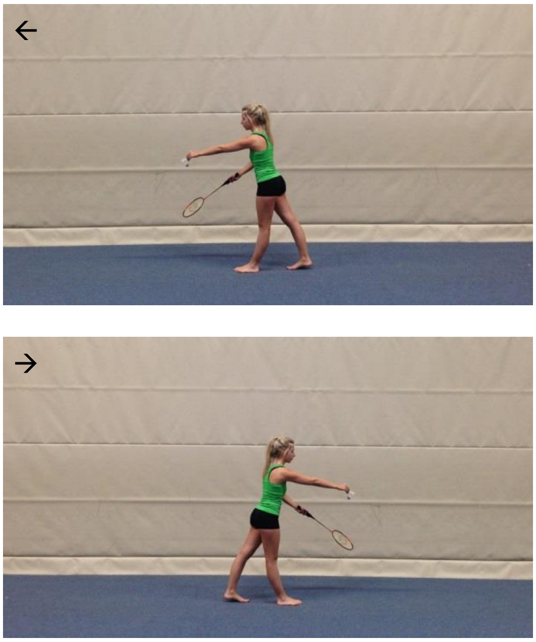

rope skipping

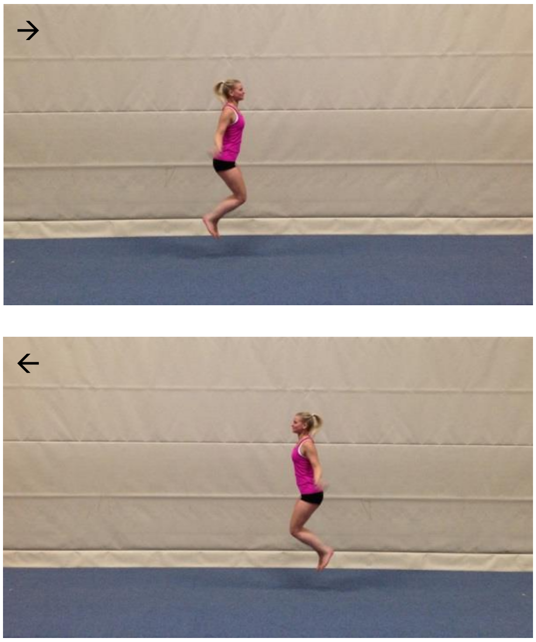

Arrows in the top left corner of pictures indicate the coding of horizontal orientation:
“→” = left-to-right; “←” = right-to-left

Experiment 1. Dynamic non-gymnastic elements.

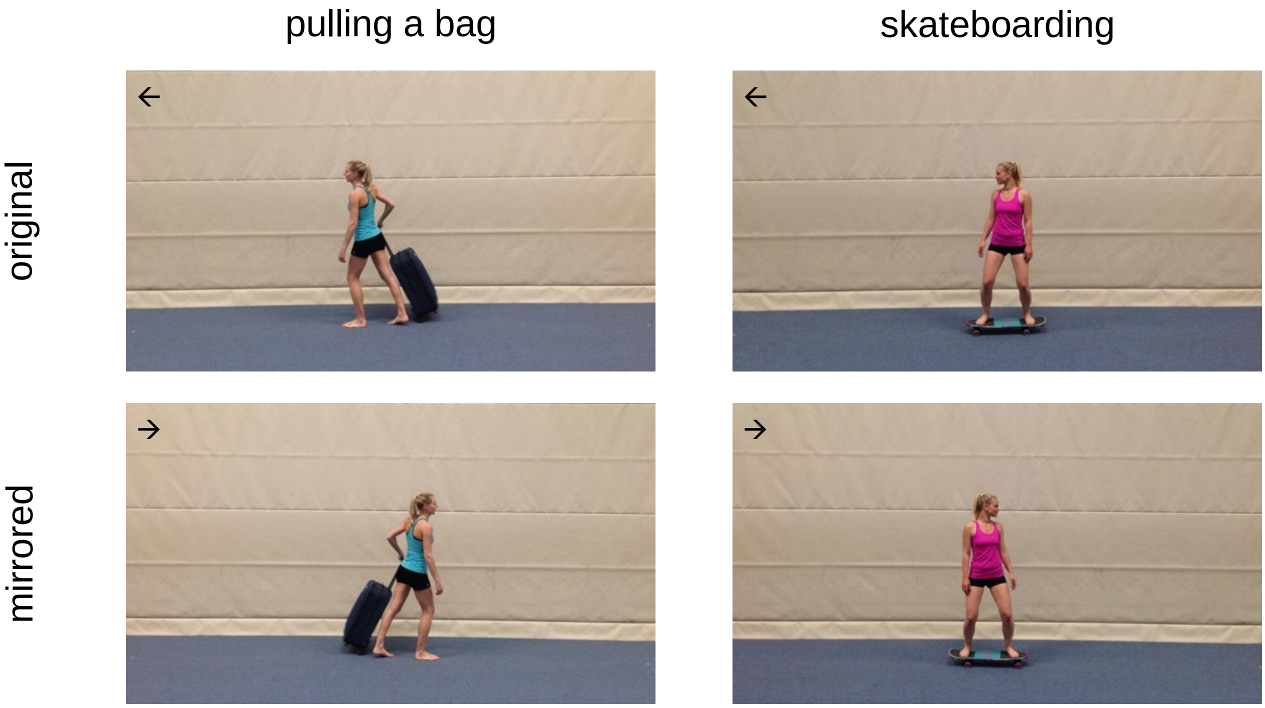

Arrows in the top left corner of pictures indicate the coding of horizontal orientation:
"→" = left-to-right; "←" = right-to-left
